# Toward the Optimization of a Perovskite‐Based Room Temperature Ozone Sensor: A Multifaceted Approach in Pursuit of Sensitivity, Stability, and Understanding of Mechanism

**DOI:** 10.1002/smll.202404430

**Published:** 2025-01-09

**Authors:** Aikaterini Argyrou, Rafaela Maria Giappa, Emmanouil Gagaoudakis, Vassilios Binas, Ioannis Remediakis, Konstantinos Brintakis, Athanasia Kostopoulou, Emmanuel Stratakis

**Affiliations:** ^1^ Institute of Electronic Structure and Laser Foundation for Research and Technology‐Hellas Vassilika Vouton Heraklion 70013 Greece; ^2^ Department of Chemistry University of Crete Vassilika Vouton Heraklion 70013 Greece; ^3^ Department of Materials Science and Engineering University of Crete Vassilika Vouton Heraklion 70013 Greece; ^4^ Department of Chemistry Aristotle University of Thessaloniki Thessaloniki 54633 Greece; ^5^ Qingdao Innovation and Development Center Harbin Engineering University Qingdao Shandong 266000 P. R. China

**Keywords:** adsorption energy, density‐functional theory, halide vacancies, mixed halide perovskites, Mn‐doped mixed halides, perovskite degradation, perovskite long‐term stability

## Abstract

Metal halide perovskites (MHPs) have attracted significant attention owing to their simple manufacturing process and unique optoelectronic properties. Their reversible electrical or optical property changes in response to oxidizing or reducing environments make them prospective materials for gas detection technologies. Despite advancements in perovskite‐based sensor research, the mechanisms behind perovskite‐gas interactions, vital for sensor performance, are still inconclusive. This work presents the first evaluation of the sensing performance and long‐term stability of MHPs, considering factors such as halide composition variation and Mn doping levels. The research reveals a clear correlation between halide composition and sensing behavior, with Br‐rich sensors displaying a p‐type response to O_3_ gas, while Cl‐rich counterparts exhibit n‐type sensing behavior. Notably, Mn‐doping significantly enhances O_3_ sensing performance by facilitating the gas adsorption process, as supported by both atomistic simulations and experimental evidence. Long‐term evaluation of the sensors provides valuable insights into evolving sensing behaviors, highlighting the impact of dynamic instabilities over time. Overall, this research offers insights into optimal halide combination and Mn‐doping levels, representing a significant step forward in engineering room temperature perovskite‐based gas sensors that are not only low‐cost and high‐performing but also durable, marking a new era in sensor technology.

## Graphical abstract

Mixed halide perovskites (MHPs), especially those doped with Mn, demonstrate a robust and sensitive response to O_3_ at room temperature. Atomistic simulations and experiments confirm that Mndoping boosts O_3_ sensing by improving gas adsorption. The evaluation of sensing performance and long‐term stability provides insights into the optimal halide combination and Mn doping level, marking a breakthrough in the development of cost‐effective, high‐performance gas sensors.

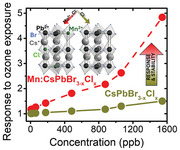

## 1 Introduction

During the past few decades, the increased industrial activity and rapid urban growth have given rise to the emission of toxic, harmful and explosive gases, contributing significantly to air pollution and posing substantial risks to both the environment and human safety.^[^ ^1^, ^2^ ^]^ As a consequence, the development of robust gas sensors, capable of detecting and monitoring ultra‐low concentrations of these hazardous compounds, is essential. Among a wide range of semiconducting gas sensing elements, metal oxide‐based sensors were the first to be reported and remain the most thoroughly studied.^[^ ^3^ ^]^ The evolution of metal oxide gas sensors over the years has been driven by continuous research, which, among others, including the influence of morphology, particle size and doping in gas‐metal oxide interactions.^[^ ^4^, ^5^, ^6^ ^]^ Recent studies have also explored stability issues related to long‐term conductivity and response, leading to the implementation of improvement strategies that have contributed to recent advances in metal oxide gas sensors.^[^ ^7^, ^8^ ^]^ However, despite the extensive research, metal oxide‐based sensors operate at high temperatures, increasing the power consumption, the overall device size and cost of gas sensors.^[^ ^9^ ^]^ Conversely, room‐temperature metal oxide‐ based sensors face challenges related to insufficient sensitivities, prolonged response/recovery times and poor reversibility.^[^ ^10^ ^]^ Therefore, there is a high demand for new materials that could overcome the former limitations.

Recently, metal halide perovskites (MHPs) with the chemical formula ABX_3_, where A is an organic or inorganic cation, B is a divalent metal and X is a halide anion, have been reported as potential gas sensing elements for the detection of a wide range of gaseous pollutants, with very promising results in terms of responsiveness and selectivity.^[^ ^11^, ^12^, ^13^, ^14^ ^]^ These sensors operate efficiently at room temperature, offering an advantage over the traditional metal oxide gas sensors, eliminating additional energy consumption and enabling the development of portable gas sensing devices.^[^ ^15^, ^16^ ^]^ The interactions between MHPs and gas molecules, which characteristically encompass charge transfer, gas‐induced defects, and defect passivation,^[^ ^17^, ^18^, ^19^, ^20^ ^]^ are inherently complex and multifaceted. Owing to this complexity, these interactions have not yet been thoroughly investigated and remain a subject of ongoing scientific inquiry.

Despite the advancements in the detection of various target gases, the field of MHP gas sensors requires further exploration into material design facets and pivotal parameters, including the impact of material composition and aging on operational efficacy. In‐depth investigations in these areas and on the underlying sensing mechanism could significantly inform the design of materials, thereby enhancing the efficiency of MHP gas sensors.

While the challenges and potential of MHPs for gas sensing are under exploration, one particular area of interest within the field of gas sensors is the detection of O_3_ pollutant, a highly reactive and oxidizing agent which is widely used in medicine and agriculture.^[^ ^21^, ^22^ ^]^ Ozone is a critical component of the Earth's atmosphere, playing a vital role in absorbing harmful ultraviolet radiation. However, at ground level, ozone is a major air pollutant with detrimental effects on human health, vegetation, and materials.^[^ ^23^, ^24^ ^]^ In recent years, an increase in the concentration of ozone gas has been observed, particularly in densely populated areas highlighting the urgent need for sensitive ozone sensors for air‐quality monitoring, assessing the effectiveness of pollution control measures, and ensuring public safety.^[^ ^25^ ^]^

Conventional ozone sensing technologies often rely on expensive and complex instrumentation, such as ultraviolet photometric detectors or chemiluminescence analyzers. These methods can be costly to implement and maintain, limiting their widespread deployment, particularly in resource‐constrained settings. Additionally, some existing sensors may suffer from cross‐sensitivity to other gases, leading to inaccurate readings.^[^ ^26^ ^]^

In response to the need for efficient ozone detection, MHPs, both hybrid organic‐inorganic and all‐inorganic, have been investigated as prospective materials. Their enhanced sensitivity and capability to operate at ambient temperatures facilitate the development of compact and lightweight sensing devices. CH_3_NH_3_PbBr_3‐x_Cl_x_ thin film was initially reported as an effective ozone sensing element, however, concerns arising from their long‐term stability and potential susceptibility upon O_3_ exposure remain unresolved.^[^ ^27^ ^]^ Contrastingly, our research group has reported the development of ultrasensitive, ligand‐free CsPbBr_3_ microcrystals characterized by markedly improved stability. Additionally, even slight morphological alterations among these microcrystals have been observed to manifest significant differences in O₃ detection responses.^[^ ^28^, ^29^ ^]^ This phenomenon was attributed to the presence of surface defects, providing insightful contributions toward clarifying the underlying sensing mechanisms.

As the charge transport in halide perovskites is predominantly facilitated by the interaction between lead and halide atoms, which significantly influence the conduction band minimum (CBM) and valence band maximum (VBM),^[^ ^30^, ^31^ ^]^ a detailed investigation into the interaction of O_3_ gas with metal cations and halide anions is crucial. Such a study is expected to offer valuable insights into the fundamental sensing mechanisms. In this context, the present research is centered on examining the sensing properties of both metal‐undoped and Mn‐doped CsPbBr_3‐x_Cl_x_ mixed halide perovskite microcrystals (µCs). The strategic incorporation of Mn as a dopant is based on its recognized role in altering the electronic properties of halide perovskites.^[^ ^32^ ^]^ Furthermore, Mn is known for its catalytic activity in decomposing O₃, suggesting its potential to enhance sensor performance.^[^ ^33^ ^]^ It is established that partial substitution of Pb with Mn within the CsPbBr_3‐x_Cl_x_ framework leads to a narrowing of the bandgap, thereby facilitating electron mobility from the valence band to the conduction band and improving both light absorption and electrical conductivity.^[^ ^34^ ^]^ Mn‐doped perovskites have also been successfully utilized as oxygen‐responsive optical probes.^[^ ^35^ ^]^ In more detail, pre‐synthesized CsPbBr_3_ µCs are modified to introduce Mn‐doping via cation exchange. Additionally, reference samples of CsPbBr_3_ and CsPbCl_3_ µCs, maintaining consistent morphology, are prepared for comparative analysis. A series of sensing experiments in response to O₃ gas exposure, complemented by atomistic simulations elucidate the preferential sites for the gas adsorption in both doped and undoped µCs by tuning the halide ratio and Mn‐doping level. This investigation contributes to understanding the influence of halide anions and metal cations on the electrical conductivity and O₃ sensitivity of MHPs. The study further monitors the temporal progression of the sensing performance, simultaneously assessing the changes in the optical, structural, and chemical properties of the sensing materials. This investigation further sheds light on the degradation pathways of all‐inorganic MHPs, thereby contributing to a deeper understanding of their long‐term stability and durability.

## 2 Results and Discussion

### 2.1 Sensing Elements’ Design: Synthesis and Features

Ligand‐free, metal‐undoped and Mn‐doped mixed halide perovskites (CsPbBr_3‐x_Cl_x_, 0<x<3) in the form of µCs with controlled anion ratio and Mn‐doping level were prepared by modifying pre‐synthesized CsPbBr_3_ µCs via room temperature cation and anion exchange processes. In the case of undoped mixed halide µCs, this was achieved through an anion exchange process, by introducing precise volume‐to‐volume ratios (v/v) of a chloride‐containing precursor (PbCl_2_) into the CsPbBr_3_ µCs solution (20%, 50%, and 80% v/v). For the Mn‐doped systems, molecular doping was performed using the same v/v of a MnCl_2_ precursor, enabling controlled Mn doping. The first method followed a conventional anion exchange approach for synthesizing mixed halide nanocrystals, while the second employed a Mn‐doping approach based on a halide exchange‐driven cation exchange (HEDCE) technique, as previously reported by Hung et al. and originally applied for the synthesis of Mn‐doped CsPb(Cl/Br)_3_ nanocrystals.^[^ ^36^, ^37^ ^]^ For comparison, CsPbBr_3_ (0% v/v) and CsPbCl_3_ (100% v/v) µCs were synthesized via a room temperature re‐precipitation‐based method by introducing a small amount of the precursor solution (CsBr/CsCl and PbBr_2_/PbCl_2_ in DMF) into the bad solvent, namely toluene (Experimental Section).

#### 2.1.1 Undoped All Inorganic Mixed Halide Perovskite µCs

**Figure 1:**
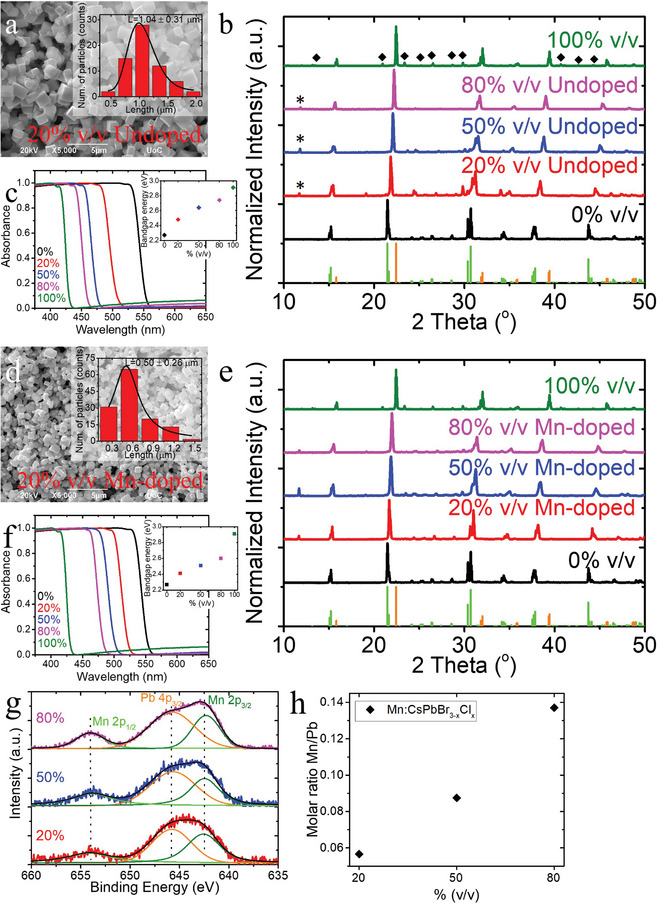
a) SEM image of 20% v/v undoped CsPbBr_3‐x_Cl_x_ µCs. The inset shows the size distribution diagram of the corresponding sample. b) XRD patterns from undoped halides with different v/v ratio and the reference samples, CsPbBr_3_ (0% v/v), and CsPbCl_3_ (100% v/v). XRD reference patterns of the orthorhombic CsPbBr_3_ crystal structure (ICDD 01‐085‐6500, light green pattern), and the tetragonal CsPbCl_3_ (ICSD#18‐0366, orange pattern) are included for comparison. The asterisks and rhombus denoted the tetragonal CsPb_2_Br_5_ (ICSD 00‐025‐0211) and monoclinic Cs_4_PbCl_6_ phase (ICSD#78‐1207), respectively. c) Absorbance spectra and bandgap energy as calculated by Tauc plot method (inset) for the same CsPbBr_3‐x_Cl_x_ samples. d) SEM image of 20% v/v Mn‐doped mixed halide µCs and corresponding inset represents the µCs size distribution diagram. e) XRD patterns of the mixed halide µCs with different volume‐to‐volume ratio and the reference CsPbBr_3_ (0% v/v). XRD reference patterns of the orthorhombic CsPbBr_3_ crystal structure (ICDD 01‐085‐6500, light green pattern), and the tetragonal CsPbCl_3_ (ICSD#18‐0366, orange pattern) are also included. f) Absorbance spectra and bandgap energy as calculated by Tauc plot method (inset) for the same samples. g) High‐resolution XPS spectra of the same samples. The deconvolution of Mn 2p reveals the presence of Mn 2p3/2 (green), Pb 4p3/2 (orange), and Mn 2p1/2 (light green). h) Mn‐to‐Pb molar ratios of Mn‐doped systems as determined by ICP‐MS.

The undoped mixed halide CsPbBr_3‐x_Cl_x_ perovskite systems exhibited a cubic‐shaped morphology as observed by scanning electron microscopy (SEM) images with an average size of ≈0.95 ± 0.12 µm, (**Figure** **1**a; Figure S1a–c, Supporting Information). These morphological features were similar to those of the pre‐synthesized CsPbBr_3_ µCs (0% v/v) used in the anion exchange process (Figure S1d, Supporting Information), as well as those of the CsPbCl_3_ µCs (100% v/v) synthesized for comparison (Figure S1e, Supporting Information). A reduction of the µC size occurred during the anion exchange process, which could be attributed to the different ionic radii of Br and Cl anions (Figure S1f, Supporting Information).

The elemental composition of the synthesized materials was estimated by Energy Dispersive Spectroscopy (EDS), as shown in Figure S2, and summarizedin the Table S1 (Supporting Information). The chemical composition of the pre‐formed CsPbBr_3_ µCs was verified by X‐ray Photoelectron Spectroscopy (XPS), corroborating the EDS results (Figures S2a and S3, Supporting Information). Specifically, the % atomic concentration, calculated from the peak areas of Cs 3d, Pb 4f and Br 3d of the XPS survey spectrum, was determined to be 22.47% Cs, 19.4% Pb, and 58.12% Br, with relative atomic concentration of Cs:Pb:Br = 1.2:1:3.1 (Figure S3, Supporting Information). Furthermore, the Bragg reflections observed in the XRD pattern of the CsPbBr_3_ µCs matched the orthorhombic crystal structure of CsPbBr_3_ (ICDD 01‐085‐6500), and no traces of secondary phases or impurities were detected (Figure 1b, black pattern). Moreover, a shift of the diffraction peaks toward higher angles was observed in the CsPbBr_3‐x_Cl_x_ µCs, indicating the gradual substitution of Br^−^ ions with Cl^−^ ions in the crystal lattice and the successful anion exchange (Figure 1b, red, blue and magenta patterns). This gradual substitution of Br^−^ with Cl^−^ ions in the crystal lattice led to its contraction which is associated with the different bond length between Pb‐Br and Pb‐Cl.^[^ ^30^ ^]^ Similar shifts of the diffraction peaks to higher angles, indicative of a cell shrinkage have been reported for CsPbBr_3_ nanocrystals with the incorporation of Cl^−^.^[^ ^32^, ^38^, ^39^ ^]^ Additionally, as the Cl concentration increases, it is noticed that the peaks between 24° to 30° gradually diminish, leading to the transformation from orthorhombic to tetragonal CsPbCl_3_ structure (Figure S4, Supporting Information). Notably, the diffraction peaks of the CsPbCl_3_ µCs matched with the tetragonal CsPbCl_3_ phase (ICSD 18–0366), with small traces of the monoclinic Cs_4_PbCl_6_ phase (ICSD 78–1207) (Figure 1b, green pattern).

The anion exchange was further demonstrated by the high‐resolution Br 3d and Cl 2p core level XPS spectra (Figure S5, Supporting Information). The binding energy of Br 3d of mixed‐halide systems exhibited a shift of ≈0.3 eV upon the introduction of Cl atoms in the CsPbBr_3_ crystal lattice, which can be assigned to the electronegativity difference between Br and Cl atoms that could affect the binding energy of Br 3d core level (Figure S5, Supporting Information). The relative intensities between Br 3d and Cs 4d decrease with increasing the Cl content, suggesting further the successful substitution of Br^−^ with Cl^−^. Moreover, a blue‐shift in the binding energy of the Pb 4f doublets with spin‐orbit splitting energy 4.9 eV was observed, indicating modifications in the chemical environment on the surface of the materials. This shift is attributed to the partial substitution of Br^−^ ions by Cl^−^ ions within the Cs‐(PbX_6_) octahedra (Figure S6, Supporting Information).^[^ ^38^, ^40^ ^]^

The successful anion exchange in the CsPbBr_3_ µCs was also confirmed by UV‐Visible (UV‐Vis) spectroscopy, revealing bandgap energy tuning as a function of the Cl‐content (Figure 1c). The absorbance spectra exhibited a clear blue‐shift of the absorption band edge with increasing Cl^−^ content, indicating an increase in the bandgap energy, which was calculated using Tauc plot method (Figure 1c inset). The bandgap energy was found to increase from 2.27 to 2.91 eV as the volume‐to‐volume (v/v %) ratio increased from 0 to 100%.

#### 2.1.2 Mn‐Doped all Inorganic Mixed Halide Perovskite µCs

The Mn‐doped CsPbBr_3‐x_Cl_x_ µCs were synthesized by a modified halide exchange‐driven cation exchange (HEDCE) approach.^[^ ^37^ ^]^ According to this method, the use of MnCl_2_ molecules was essential for achieving simultaneous halide and cation exchange within the same lattice site. The cubic‐shaped morphology of the µCs remained unchanged during the cation exchange process, even after increasing the volume of the Mn‐containing precursor (Figure 1d; Figure S7, Supporting Information). However, the µC size was reduced from 0.95 ± 0.12 to 0.49 ± 0.02 µm (Figures S1 and S7, Supporting Information), while remaining almost unchanged with further increase of the Mn‐precursor volume. The difference in ionic radii between Br and Cl anions, as well as the Pb and Mn cations, may contribute to the observed size reduction, however, other contributing factors, such as defect‐mediated dissolution in DMF, cannot be excluded, where the incorporation of Mn could introduce defects in the crystal structure, making it more susceptible to dissolution. The constant size for varying the Mn‐doping level could be due to the small percentage of the Mn‐inserted cations. The elemental composition was estimated by EDS (Figure S8 and Table S2, Supporting Information) while the cation and anion exchange upon the addition of the MnCl_2_ precursor were confirmed by the XRD patterns and the XPS spectra. Similar to the undoped systems, the XRD patterns of Mn‐doped systems demonstrated a monotonic shift toward higher angles with increasing the amount of MnCl_2_ precursor (Figure 1e). This shift was attributed to both the substitution of Br^−^ with Cl^−^ anions and Pb^2+^ with Mn^2+^ cations. Furthermore, similar to the undoped systems, Mn‐doped CsPbBr_3‐x_Cl_x_ exhibited comparable XPS spectral characteristics attributed to anion exchange phenomena (Figures S9 and S10, Supporting Information). The gradual increase in Mn^2+^ concentration in the CsPbBr_3‐x_Cl_x_ µCs upon increasing the volume percentage was also confirmed by the enhanced relative intensity of Mn 2p_3/2_ peak compared to Pb 4p_3/2_ peak in high‐resolution XPS spectra (Figure 1g). Nevertheless, the molar concentration of the Mn in the Mn‐doped µCs was determined by ICP‐ΜS as the quantification of Mn^2+^ from the XPS survey spectra proved challenging due to the complexity of deconvoluting the Mn 2p peaks, given the overlapping of Pb 4p_3/2_ peak interference (Figure 1h).

Similar to the undoped mixed halide systems, the absorbance spectra of the Mn‐doped sensors showed a blue‐shift of the band edge as the volume‐to‐volume ratio was increased, resulting in the bandgap energy increase (Figure 1f). This rise was originated from the synergetic combination of the increase in the Cl‐to‐Br molar ratio in the CsPbBr_3_ host lattice but also and to the incorporation of Mn^2+^. The latter contribution, according to the literature in similar systems, causes slight blue shifts of the absorbance spectra and may be attributed to alloying effects on the perovskite band structure.^[^ ^41^ ^]^

Moreover, despite maintaining consistent ratios of PbCl_2_ and MnCl_2_ precursor solutions at 20%, 50%, and 80% v/v and the same reaction times for the fabrication of undoped and Mn‐doped CsPbBr_3‐x_Cl_x_ µCs, variations in halide content were observed (Figure S11a, Supporting Information). The Br content decreased as the volume‐to‐volume ratio increased, and conversely, an increase was observed for the Cl content for both undoped and Mn‐doped µCs. However, the Cl‐content was lower in the Mn‐doped systems across all volume‐to‐volume ratios. These variations may be attributed to the simultaneous anion and cation exchange, and the choice of MnCl_2_ as an anion exchange precursor. The [PbBr_6_]^4^^−^ octahedra open up via extended halide exchange including also the partial substitution of Pb sites. This process occurs at longer reaction times than in the undoped systems due to the difficulty of the molecule MnCl_2_ to diffuse into the crystal lattice.^[^ ^32^, ^37^ ^]^ This finding was further supported by comparing the absorbance spectra and the bandgap energies of the undoped and Mn‐doped systems (Figure S11b,c, Supporting Information). Contrary to the anticipated larger blue shifts and higher bandgap energies for the Mn‐doped systems, the opposite was observed suggesting a slower diffusion of MnCl_2_ compared to PbCl_2_.

### 2.2 Ozone Sensing Performance of All‐Inorganic Metal Halide Perovskite µCs

The precipitated perovskite µCs were deposited onto commercial interdigitated platinum electrodes supported on glass substrate and left to dry under vacuum. Subsequently, the ozone detection capability of all fabricated perovskite‐based sensors was assessed through room‐temperature electrical measurements under total dark operating conditions. The gas sensing experiments were carried out in a home‐made chamber with a constant pressure of 700 mbar whereas a voltage of 2 to 3.5 V was applied. The sensing process was initiated by exposing the sensors to ozone gas for 150 s, followed by a 200 s recovery with synthetic air. All samples were exposed to different ozone concentrations ranging from 1567 down to 6 ppb (Experimental Section).

#### 2.2.1 Ozone Sensing Performance of Undoped Mixed Halide Perovskites

**Figure 2:**
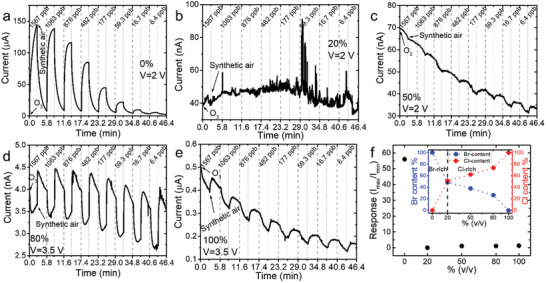
a–e) O_3_ sensing performance of the undoped perovskite‐based sensors, by varying the volume‐to‐volume ratio from 0 to 100%. f) Calculated response of the undoped sensing systems at 1567 ppb.The halide content of the undoped µCs as calculated by XPS spectra is shown as an inset.

The electrical response diagrams of each sensor upon O_3_ exposure revealed the significant influence of the halide composition in the sensing process (**Figure** **2**). Notably, the undoped sensors with 5 and 80% v/v exhibited a decrease in current intensity upon O_3_ exposure, followed by an increase upon synthetic air (Figure 2c,d). This trend was observed for all tested O_3_ concentrations, ranging from 1567 ppb down to 4 ppb, indicating an n‐type sensing behavior, akin to that observed in CsPbCl_3_ µCs (Figure 2e). Conversely, the 0% v/v CsPbBr_3_ based‐sensor displayed a contrasting response pattern, and particularly, the current was increased to a maximum current value upon O_3_ exposure, followed by a recovery to its baseline in the presence of synthetic air, suggesting a p‐type behavior (Figure 2a), as has been previously shown by our group.^[^ ^28^, ^29^ ^]^ By correlating these results with the halide content diagram obtained from the XPS spectra (Figure 2f inset; Figure S12, Supporting Information), it was observed that the sensors with higher Br content (Br‐rich) displayed a p‐type behavior, while the Cl‐rich ones exhibited n‐type sensing behavior. This conclusion was further supported by the sensing behavior of the Br‐rich 10% v/v sensor, which was found to be analogous to the CsPbBr_3_‐based sensor (Figure S13, Supporting Information). Notably, the 20% v/v perovskite‐based sensor, which had nearly equimolar halide content, was unable to detect O_3_ at any concentration, as the competing p‐ and n‐type sensing behaviors intersected, resulting in the absence of O_3_ sensing behavior (Figure 2b).

Moreover, the response calculated for the highest O_3_ concentration showed that the mixed halide perovskite‐based sensors maintain a nearly constant response as the volume‐to‐volume ratio increases, which was similar to that of the CsPbCl_3_ sensor, but considerably lower than that of the CsPbBr_3_ sensor. This observation indicated that sensors with higher Br content exhibited higher response, which decreased with the incorporation of Cl^−^ ions within the crystal lattice (Figure 2f).

#### 2.2.2 Ozone Sensing Performance of Mn‐Doped Mixed Halide Perovskites

**Figure 3:**
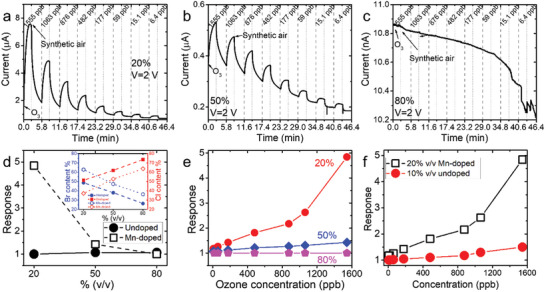
O_3_ sensing performance of the Mn‐doped a) 20%, b) 50%, and c) 80% v/v perovskite‐based sensors. d) Calculated response of Mn‐doped (white circle) and undoped (black circle) perovskites at 1567 ppb O_3_ concentration. The halide content of the Mn‐doped µCs calculated by XPS spectra is shown as an inset. e) Response of the Mn‐doped sensors varying the Mn‐doping level as a function of O_3_ concentration. f) Response comparison of the 10% v/v undoped (red circle) and 20% v/v Mn‐doped sensor (white circle) as a function of O_3_ concentration.

**Figure** **3** illustrates the performance of Mn‐doped CsPbBr_3‐x_Cl_x_ perovskite‐based sensors upon O_3_ exposure. Intriguingly, the 20 and 50% v/v sensors demonstrated a p‐type behavior in response to O_3_ gas (Figure 3a,b), while the 80% v/v Mn‐doped sensor showed no sensing capability (Figure 3c), contradicting the expected n‐type behavior associated with its higher concentration of Cl relative to Br ions. This observation suggests a potential overlap in the roles of Mn and Br ions with the influence of Cl ions in the overall sensing behavior. These results contrast with the corresponding undoped mixed halide sensors, which exhibited an n‐type behavior for the 50% and 80% volume‐to‐volume ratios, and no sensing capability for the 20% v/v sensor (Figure 2b–d). Notably, the Mn‐doped perovskite‐based sensors showed an improved response at 1567 ppb compared to their undoped counterparts, with the maximum improvement for the 20% v/v Mn‐doped sensor (Figure 3d). This sensor also exhibited the highest response among all Mn‐doped sensors across all O_3_ concentrations (Figure 3e). These results indicated that Mn‐doping has a significant and specific role on the sensing behavior, but it was not easily distinguished from that of the halide contribution. As mentioned previously, sensors with the same volume‐to‐volume ratio do not have the same Br to Cl ratio (Figure 2d inset; Figure S14, Supporting Information).

To assess the impact of Mn‐doping on sensing performance, a comparison in the sensing behavior between Mn‐doped and undoped systems with similar Br‐to‐Cl ratio was essential. Notably, both 20% v/v undoped and 50% v/v Mn‐doped‐based sensors demonstrated nearly equimolar halide content, however, significant variations in the sensing performance were observed (Figure 3d inset). Particularly, the 20% v/v undoped sensor did not exhibit any sensing capability, as previously discussed (Figure 2b), while the 50% v/v Mn‐doped based‐sensor showed a p‐type sensing behavior (Figure 3b), suggesting that Mn‐doping can override the synergetic effect of the halides. Moreover, the influence of Mn on the sensing performance was evident in the 50% v/v undoped and 80% v/v Mn‐doped based systems demonstrating equimolar halide content as well. The strong effect of Mn‐doping was further studied by comparing the response between the 10% v/v undoped (Figure S13, Supporting Information) and the 20% v/v Mn‐doped sensors (Figure 3a). Given that both sensors are characterized by higher concentration of Br compared to Cl ions, a p‐type behavior was observed, as expected (Figure 3a; Figure S13, Supporting Information). However, while both systems were capable of detecting low ozone concentrations, the Mn‐doped system displayed a capability to effectively detect ultra‐low gas concentrations below 15 ppb (Figure 3a) in contrast to the undoped one (Figure S13, Supporting Information). The calculated response of the 20% v/v Mn‐doped system surpassed that of the 10% v/v undoped counterpart by more than three times (Figure 3f).

### 2.3 Ozone Sensing in Mixed Halide Perovskites Through Site‐Specific Analysis and Computational Modeling

A thorough understanding of the ozone sensing mechanism in MHPs requires identifying the specific sites where O_3_ molecules interact with the perovskite surface. Previous studies have identified halide vacancies and trap states as critical sites for gas adsorption/desorption, a phenomenon substantiated by observable alterations in the material's electrical conductivity. However, it is noteworthy that only a singular study examining the sensing behavior of a perovskite film in the presence of oxygen has corroborated these findings with theoretical calculations.^[^ ^19^, ^27^, ^42^, ^43^ ^]^

In general, a good sensing material could offer strong binding to the target gas while it could greatly alter its electronic structure upon adsorption. Both these properties can be probed using DFT simulations, which can provide accurate adsorption energies, which is a measure of the strength of the gas‐solid interaction, and density of states, which offers insights on the change of electronic properties of the material during sensing (Section S2, Supporting Information). To elucidate the sensing properties of these perovskite materials, DFT simulations have been employed for a series of model systems. Simplified surface models and O_2_ molecules instead of O_3_ have been used for computational efficiency, as prior DFT investigations focused on Ο_3_ sensors have indicated that adsorbed O_3_ predominantly appears as either oxygen molecules or atoms. The binding of O_2_ and O_3_ to polar surfaces relies on electron charge transfer toward surface atoms and the coupling of oxygen's s and p orbitals with the s and p orbitals of an electropositive surface atom.^[^ ^42^, ^44^, ^45^ ^]^

Τhe (001) surface of the CsPbBr_3_ has been utilized as the model surface, owing to its demonstrated stability, corroborated previously by the findings of Xing et al.^[^ ^42^ ^]^ Moreover, since halide‐ and metal‐atom vacancies can introduce trap states and act as active sites for gas molecules' absorption/desorption upon O_3_ exposure, vacancies have been introduced in the models.

**Figure 4:**
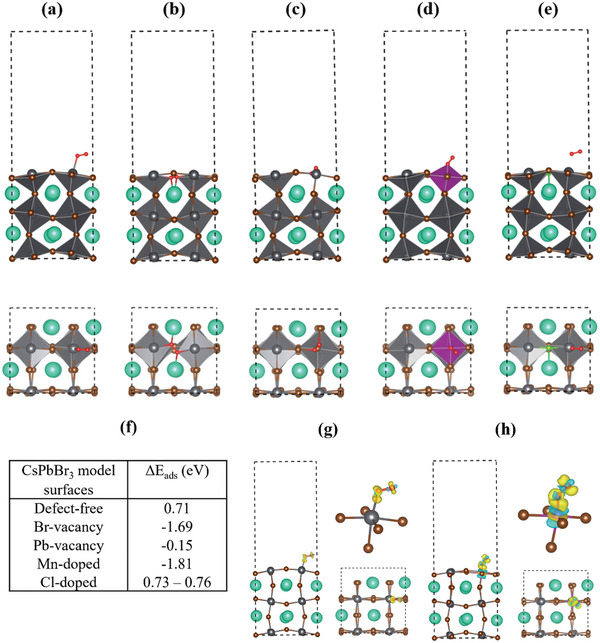
DFT‐optimized configurations of the model surfaces viewed along b (upper line) and c (bottom line) axis; a) defect‐free CsPbBr_3_, b) CsPbBr_3_ with Br vacancy and c) with Pb vacancy, d) Mn‐doped and e) Cl‐doped CsPbBr_3_ slabs. Cs, Pb, Br, Mn, Cl, and O atoms are illustrated as cyan, gray, brown, magenta, green, and red atoms, respectively. Dashed lines indicate the periodic boundaries of the simulation supercell. f) Adsorption energies of O_2_ adsorbed on the defect‐free (001) surface of CsPbBr_3_, at the Br and Pb vacancies, and at the Mn‐ and Cl‐ doped modified surfaces. The range of values for the Cl‐doped system corresponds to the different Cl concentrations examined. g,h) Charge density difference plots calculated at 0.005 (e/bohr^3^) isosurface for g) O_2_ adsorbed on the defect‐free and h) Mn‐doped CsPbBr_3_ surface, viewed along the b (side view) and c (top view) axis, and magnified at the corresponding adsorption sites at the upper right plots. Yellow and cyan areas illustrate charge accumulation and depletion, respectively.

The effect of anion exchange and cation doping, as well as the formation of defects were investigated using DFT by considering different 5‐layer surface models (**Figure** **4**a–e); a) without defects, b) with Br vacancy in the outmost surface layer, c) with Pb vacancy in the outmost surface layer, d) with Mn doping, where 1/4 of the outmost surface Pb atoms are substituted by Mn and e) with Cl doping, where 1/8 of the outmost surface atoms are substituted by Cl. For case e), systems with higher Cl concentrations as well as systems with Cl vacancies were also employed (see additional models in Section S2.1, Supporting Information).

The calculated absorption energies of the above models (Figure 4f; and Table S3, Supporting Information) showed that the O_2_ adsorption was energetically more favorable at Br‐vacancies (ΔE_ads_ = ‐1.69 eV) compared to the defect‐free surface (ΔE_ads_ = 0.71 eV) and Cl‐doped surface (ΔE_ads_ = 0.73 eV). This indicates that oxygen binding is expected to occur primarily close to halide vacancies, as the adsorption energies for the defect‐free case suggest minimal interaction between molecular oxygen and the surface. This absence of interaction was further observed for the Cl doped surface with all Br atoms in the uppermost layer replaced by Cl atoms, as well as for the CsPbCl_3_ surface (ΔE_ads_ = 0.75 and 0.76 eV, respectively), (Figure S15b,c, Supporting Information). However, similar to the CsPbBr_3_ surface with Br‐vacancy, molecular oxygen was found to bound strongly in the Cl‐vacancy site of the defected CsPbCl_3_ surface (ΔE_ads_ = ‐1.87 eV) (Figure S15d, Supporting Information). It should be noted that the positive binding energy of the oxygen molecule on the defect‐free perovskite surfaces was a result of the change in the electron configuration of the molecule upon adsorption from paramagnetic to diamagnetic.

Similar to Br‐vacancies, Mn sites in the Mn‐doped surfaces were found to bound strongly molecular oxygen, exhibiting an even greater enhancement of the binding energy (ΔE_ads_ = ‐1.81 eV), thus contributing further to the gas sensing response enhancement. This is a remarkable finding, indicating that Mn doping alone could introduce oxygen binding sites comparable in strength to halide vacancies. To better understand this enhancement, charge density difference plots were calculated for the defect‐free and the Mn‐doped surfaces, visualizing the changes in the charge density of these surfaces upon interaction with oxygen (Figure 4g,h). As it can be clearly seen, Mn‐doped surface exhibited stronger polarization of the oxygen electron cloud and a more prominent polarization of the charge density of the Mn atoms. This stronger polarization effect of Mn compared to Pb, followed the fact that Mn atoms are less electronegative than Pb, thus having a larger electronegativity difference with oxygen atoms. Accordingly, the Mn─O bond is more polar than P─O, a fact also reflected in bond lengths, with *d*_*Mn* − *O*_ and *d*_*Pb* − *O*_ being 1.71 and 2.61 Å, respectively.

The introduction of specific impurities, either by adding electron‐rich or electron‐deficient sites in a semiconductor modifies its band structure by introducing energy levels associated with the dopants. To gain a more comprehensive understanding on the impact of the different doping scenarios on the electrical conductivity and O_3_ sensing behavior, the electronic band structure (BS) and the partial density of states (PDOS) were calculated for all model cases under study (Figures S16–S22 and Sections S2.1 and S2.2 all the details for the calculations, Supporting Information). Minimal electronic structural changes were caused by bromide substitution with chlorine, maintaining the defect‐free surface's characteristics, as chlorine's states lie deep within the band structure. However, increasing chlorine substitution amplifies its valence band contribution, nudging the Fermi level toward n‐type doping characteristics. On the other side, new donor states in the conduction band and localized d orbital states within the bandgap were introduced by manganese doping. Upon adsorption of oxygen on Mn‐doped model surface, the shallow donor states were quite withdrawn, and new acceptor states appeared at the top of the valence band. The d‐states of Mn remained localized at the same relative position within the gap, while the oxygen states exhibit a non‐negligible density located both at the valence band and close to the conduction band minimum.

It should be noted that the calculation of fine details of the electronic structure, such as the precise position of impurity levels, should be handled with caution as it could be affected by the employed models. For example, slab‐based simulations mimic surface doping and not bulk doping, with the former possibly resulting in localized electronic states on the surface that can alter the electronic structure.^[^ ^46^ ^]^ On the other hand, DFT simulations effectively explain both the strong effect of Mn doping on adsorption energy and the changes in the electronic structure of all systems upon oxygen adsorption, as in all cases oxygen alters dramatically the electronic states.

Mn doping significantly impacts two key properties for ozone sensing: the adsorption energy of oxygen species and the electronic band structure of the material. Mn doping has an effect similar to that of surface halogen vacancies, increasing the adsorption energy compared to the defect‐free surface. This finding aligns with the experimentally observed enhancement of O_3_ sensing performance upon Mn doping. Additionally, Mn doping introduces new electronic states within the bandgap, potentially influencing the charge transport properties and contributing to the enhanced sensing behavior. Overall, the DFT calculations provide valuable insights into the mechanism of O_3_ sensing and highlight the role of Mn doping in improving sensor performance.

### 2.4 Evaluation of the Sensing Capability and Material Stability of Lead Halide Perovskite‐based‐Sensors Over Time

The sensing capabilities of each sensor were re‐evaluated after a month of storage under ambient conditions, referred to as aged sensors. Interestingly, both undoped and Mn‐doped mixed halide perovskite‐based sensors, as well as the pure CsPbBr_3_ and CsPbCl_3_ µCs exhibited a conductivity improvement over time (Figures S23–S25, Supporting Information). Notably, the undoped 20% v/v sensor showed a slight enhancement upon O_3_ exposure over time, compared to its initial absence of a sensing signal (Figure S24a, Supporting Information), while the most prominent improvement over time was observed in the 80% v/v Mn‐doped sensor, which initially displayed no sensing capability (Figure S24c, Supporting Information). Additionally, the sensing behavior (p‐ or n‐type) was preserved after storage under ambient conditions for all the sensors, except for the 50% v/v undoped mixed‐halide sensor, which demonstrated an n‐type to p‐type conductivity transition upon O_3_ exposure over time (Figure S24b, Supporting Information).

**Figure 5:**
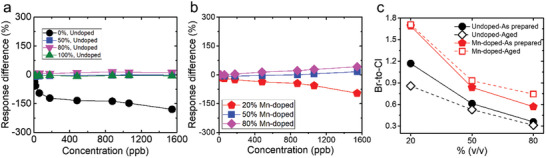
Percentage response difference between the as prepared and aged a) undoped and b) Mn‐doped perovskite‐based sensors as a function of the O_3_ concentration. c) Br‐to‐Cl ratio of as prepared (filled symbols) and aged (hollow symbols) undoped (circles) and Mn‐doped (pentagons) perovskites.

Furthermore, a comparison between the initial response and that of the aged undoped sensors for various O_3_ concentrations revealed that all systems displayed a fairly stable response over time (Figure S27, Supporting Information), with a percentage response difference close to zero (**Figure** **5**a and Equation (2)). This behavior is similar to that observed for 100% v/v CsPbCl_3_ sensor (Figure 5a; Figure S26b, Supporting Information).

On the other hand, Mn‐doped sensors showed instabilities in their response over time. The 20% v/v Mn‐doped sensor exhibited a lower response upon aging, indicating deterioration (response_aged_ < response_as prepared_), (Figure S28a, Supporting Information). Conversely, the 50% v/v Mn‐doped system remained quite stable with nearly zero percentage response difference (Figure 5b, blue points; Figure S28b, Supporting Information).Tthe 80% Mn‐doped sensor, however, showed a significant improvement with a positive response difference (Figure 5b magenta points; Figure S28c, Supporting Information). These results suggest a correlation between the response stability over time and the halide content and doping level.

It is noteworthy that, compared to CsPbBr_3_‐based sensor which exhibited a significant response decrease upon time with a negative percentage difference of 150% (Figure 5a, black points; Figure S26a, Supporting Information), both undoped and Mn‐doped‐ based mixed halide sensors appeared more stable in terms of O_3_ response. Although the initial observation might suggest an advantage for the pure CsPbCl_3_‐based sensor in terms of response and stability (Figure 5a; Figure S26b, Supporting Information), compared to the mixed halide perovskite‐based sensors (Figures S27 and 28, Supporting Information). However, closer examination revealed its inability to distinguish different ozone concentrations, as evidenced by the similarity in oxidation curves (Figure S23b, Supporting Information). Notably, the 50% v/v Mn‐doped sensor emerged as the sensor with the optimal combination of the highest response and stability among all the tested sensors (Figure 5b; Figure S28b, Supporting Information). Despite having similar a Br‐to‐Cl ratio to the 20% v/v undoped sensor, as previously discussed (Figure 2b), this comparison provides clear evidence that Mn‐doping improves response and sensing stability.

To understand the variations in the sensing behavior of both undoped and Mn‐doped perovskite‐based sensors over time (as‐prepared and aged), a series of repetitive structural and morphological characterizations were carried out. Starting from the undoped perovskite sensors, SEM imaging revealed a well‐preserved surface morphology of all the µCs over time (Figure S29, Supporting Information), while the XRD patterns and the absorbance spectra (Figures S30–S31, Supporting Information) revealed no substantial difference between the as‐prepared and aged samples. However, a decrease in the Br‐to‐Cl ratio, determined by XPS survey spectra, was observed in all the aged samples compared to the as‐prepared ones (Figure 5c; Figure S32, Supporting Information), indicating the formation of Br‐vacancies on the surface of the materials during the aging process. The most significant difference in Br‐to‐Cl ratio between the as‐prepared and aged sensor was observed for the 20% undoped sensor (Figure 5c), confirming that this system exhibits the lowest stability over time (Figure S27a, Supporting Information).

Similarly, CsPbBr_3_ µCs showed a reduction in the Br‐to‐Pb ratio by 4.3% compared to the initial composition, indicating Br deficiency, related to the formation of Br vacancies on the material' surface over time (Figure S33a, Supporting Information). Notably, halide vacancies are the most mobile charges in halide perovskites due to their low migration activation energies. As a result, they tend to migrate to the surface over time.^[^ ^47^, ^48^ ^]^ This migration may induce ion movement, which potentially contributes to the observed increase in conductivity when voltage is applied, explaining the increased current intensity after exposing the perovskites to ambient conditions.^[^ ^49^, ^50^, ^51^ ^]^ In contrast, CsPbCl_3_ µCs showed a 4.5% increase in the Cl content on the surface of the material over time (Figure S33b, Supporting Information). This redistribution of Cl on the surface of the µCs could explain enhanced conductivity of these µ, while the absence of extra vacancies on the surface could explain the stability of the response of this sensor with aging.

To further assess the fine structure of specific peaks of interest of the undoped systems, XPS narrow scan were conducted. In particular, Cs 3d, Pb 4f, and Br 3d chemical states were investigated (Figures S34–S37, Supporting Information). After exposing the samples to ambient conditions for one month, no change in Cs 3d peaks were detected in all undoped perovskite systems indicating the low bonding interactions of Cs with Br and Cl ions either on surface or crystal lattice (Figure S34a, Supporting Information).^[^ ^52^ ^]^ Similarly, no change was observed for both CsPbBr_3_ and CsPbCl_3_ µCs (Figure S34b,c, Supporting Information).

Furthermore, the f_7/2_ and f_5/2_ chemical states of Pb 4f were deconvoluted into doublets as depicted in Figure S35 (Supporting Information). The peaks at ≈138.1 and 143 eV correspond to the Pb^2+^ species originating from Pb‐X bonds, while no traces of metallic Pb^0^ were observed, even after the prolonged exposure of the µCs to ambient conditions (Figure S35a, Supporting Information). Additionally, a red‐shift of Pb 4f doublets in the binding energy of 20% v/v undoped perovskite µCs was observed, implying modifications in the local environment of [PbX_6_]^[^ ^4^ ^]4−^ octahedral (Figure S35c, Supporting Information). These changes may arise from the length of Pb‐X bonds, which are associated with the strength of Pb‐X interactions, possibly originating from halide migration or the formation of surface defects.^[^ ^38^, ^53^, ^54^ ^]^ In contrast, the binding energy in Pb 4f of 50% and 80% v/v undoped µCs and CsPbCl_3_ did not display any significant variations over time (Figure S35b,c, Supporting Information), indicating the high stability of Cl‐rich species toward ambient air and light, a finding in accordance with previous predictions by Cai et al.^[^ ^55^ ^]^ Similar trends were observed in Br 3d and Cl 2p spectra (Figures S36 and S37, Supporting Information). In particular, it was noticed that the undoped systems exhibited a decrease in the relative intensity between Br 3d and Cs 4d peaks, suggesting further the reduction in Br content on the surface of each sensor and possibly the formation of Br vacancies (Figure S36, Supporting Information). It is noteworthy that among all the undoped systems the 20% v/v exhibited a more pronounced decrease compared to the other two, while the 50% v/v showed a more substantial decrease than the 80% v/v, indicating a concentration‐dependent effect (Figure 5c). This observation is in consistent with the decreased Br‐to‐Cl ratio over time and the change in the sensing behavior of 20% and 50% v/v sensors toward p‐type conductivity over time.

**Figure 6:**
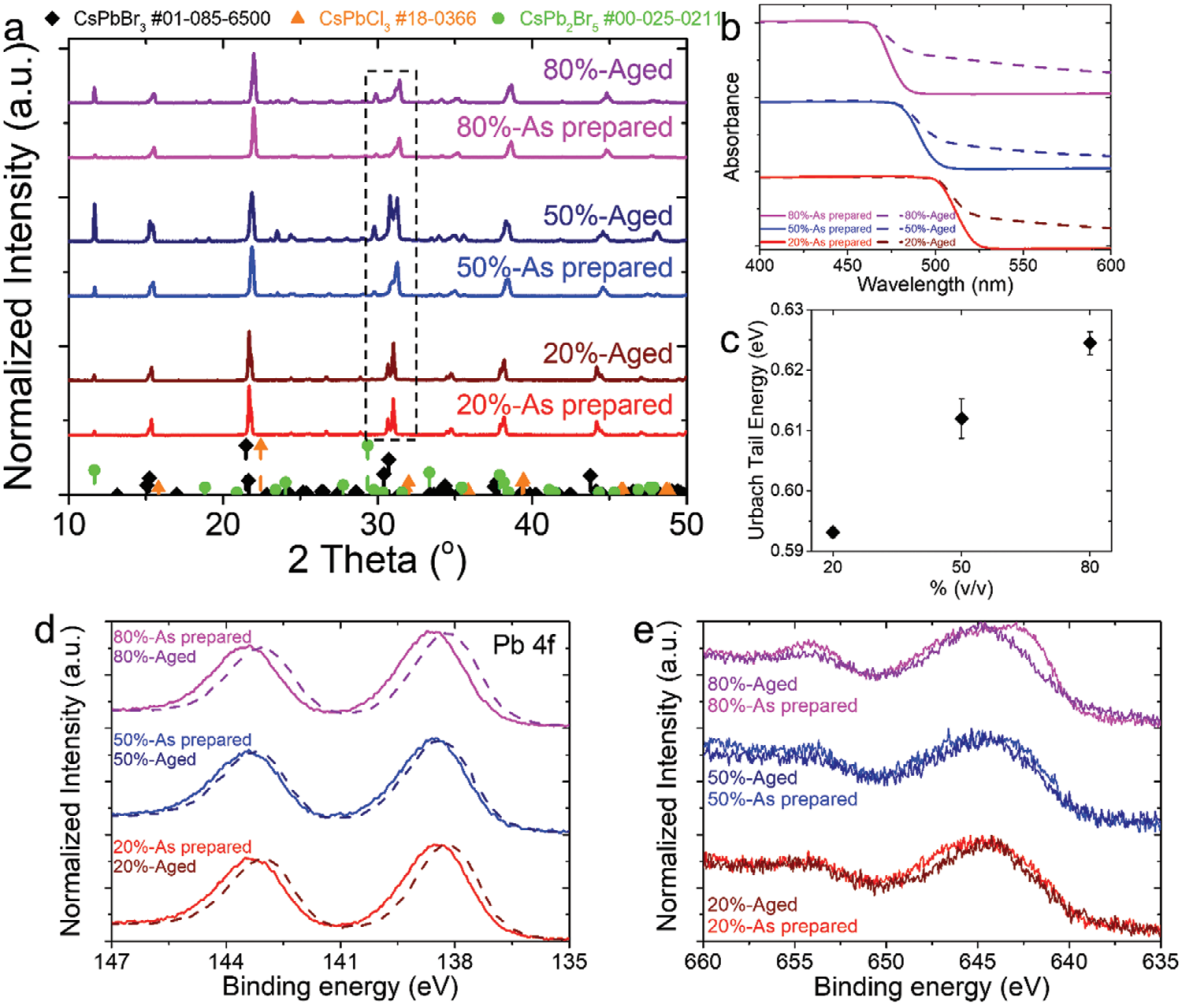
a) XRD patterns of the as prepared and aged Mn‐doped mixed halide perovksite µCs with varying v/v ratio after exposure to ambient conditions for one month. b) Absorbance spectra of the as‐prepared (solid lines) and aged samples (dashed lines). c) Calculated Urbach energy as a function of volume‐to‐volume ratio. d,e) High‐resolution XPS spectra of Pb 4f and Mn 2p respectively.

In the case of Mn‐doped systems, SEM imaging revealed that the µCs retain their morphological features (Figure S38, Supporting Information), however significant differences were observed both structurally and optically. Particularly, the XRD analysis revealed an increase in the intensity of the secondary non‐perovskite CsPb_2_X_5_ byproducts over time, which increased with increasing the volume‐to‐volume ratio (Figure S39, Supporting Information). Apart from the formation of impurities after the prolonged exposure of the Mn‐doped sensors to ambient conditions, a careful examination of the XRD patterns revealed peak splitting at ≈15.5°, 21.8^ο^, 31.3°, and 35° (**Figure** **6**a), which indicated symmetry‐breaking and subsequently the phase transformation from their initial sub‐tetragonal phase into the more energetically favorable orthorhombic crystal structure due to the incorporation of Cl anions.

Interestingly, the structural properties of both Mn‐doped systems were re‐evaluated after one year revealing more pronounced peak splitting for all samples with different v/v ratios (Figure S39, Supporting Information). Previous studies suggested that this transition resulted from the relatively soft crystal lattice of these materials, which caused intrinsic disorder phenomena, such as vacancy and dislocation formation.^[^ ^56^, ^57^ ^]^ These structural imperfections render halide perovskites highly sensitive to external stimuli such as light, water, and oxygen.^[^ ^58^, ^59^ ^]^ Consequently, this susceptibility accelerated the formation and migration of ion defects, resulting to the distortion of crystal lattice by impacting the tilting of [PbΧ_6_]^4−^ octahedra and the subsequent reduction of its symmetry.^[^ ^60^, ^61^ ^]^ This finding further corroborated the interaction of CsPbX_3_ µCs with atmospheric species and therefore the formation of CsPb_2_X_5_ impurities (Figure 6a). Additionally, regarding the Mn‐doped systems, the introduction of dopants accelerated the deterioration process, possibly due the large radius mismatch between Pb^2+^ and Mn^2+^.^[^ ^62^ ^]^

Further insights into the effects of the prolonged exposure to ambient conditions on the Mn‐doped systems were obtained through UV–vis spectroscopy (Figure 6b). The absorbance spectra of the Mn‐doped systems revealed an exponential decrease with decreasing energy over time, which can be described by the Urbach rule equation.^[^ ^63^ ^]^ Calculation of Urbach energy showed an increase with higher doping level (Figure 6c). This Urbach tail could be linked to the presence of localized states in the bandgap, caused by imperfections within the crystal lattice related to dynamic disorder.^[^ ^64^ ^]^ Such dynamic disorder makes halide perovskites susceptible to intrinsic defects, inducing structural instabilities that were previously confirmed previously by XRD (Figure 6a). These instabilities could arise from the redistribution of Mn dopants over time, leading to changes in the electronic structure and absorbance of the materials. Alternatively, they could result from degradation caused by exposure to various environmental factors, including photoxidation, which is known to influence the stability of Mn‐doped nanocrystals.^[^ ^32^ ^]^

The binding energies of Cs, Pb, Br and Cl were also examined to determine surface alterations that occurred in each Mn‐doped perovskite material over time. Similar to the undoped systems, Mn‐doped mixed halide perovskite µCs did not exhibit any chemical changes in Cs 3d doublets, however a red‐shift of Pb 4f and Cl 2p doublets in the binding energy was observed for all Mn‐doped systems (Figure 6d; Figures S40 and S41, Supporting Information). Moreover, even though no changes in the relative intensities between Br 3d and Cs 4d peaks were observed over time, an increasing Br‐to‐Cl ratio was found with increasing the Mn‐content, indicating the possible formation of Cl vacancies (Figure 6c; Figures S42 and S43, Supporting Information). Of particular interest is the remarkable increase in the Br‐to‐Cl ratio observed in the 80% v/v Mn‐doped system, compared to the 20% and 50% counterparts, aligning with the significant increase in response over time (Figure 6c). Additionally, Mn 2p narrow scans revealed a decrease of Mn^2+^ on the surface of Mn‐doped systems after prolonged exposure to ambient conditions, as shown in Figure 6e. This observation, along with the formation of Cl vacancies on the surface of the µCs, suggests that MnCl_2_ may have migrated deeper into the crystal lattice through a defect‐mediated diffusion, potentially affecting stability and ionic conductivity. It is noteworthy that, despite the observed instabilities of Mn‐doped mixed halide perovskites over time, the Mn‐doped perovskite‐ sensors retained the capability to detect O_3_. Additionally, despite the deterioration, the 50% v/v Mn‐doped based‐sensor demonstrated the best response stability over time. Therefore, while this sensor exhibits low absolute response, its enhanced stability and prolonged operational lifetime could be advantageous in applications emphasizing durability and consistent performance.

## 3 Conclusion

This study highlights the critical role of metal cations and halide anions in O_3_ detection, revealing that ligand‐free undoped, and Mn‐doped mixed halide CsPbBr_3‐x_Cl_x_ perovskites exhibit varying responses based on halide content, with bromine‐rich sensors showing p‐type behavior, while chlorine‐rich ones exhibit n‐type behavior. Surface defects, particularly bromine and chlorine vacancies, are vital for oxygen adsorption and O_3_ sensing, as revealed by DFT calculations. In addition, Mn‐doping enhances sensor performance by facilitating the O_3_ absorption process. Interestingly, deviations in sensing behavior are observed in Mn‐doped systems compared to the undoped based sensors, suggesting that Mn and Br ions may play overlapping roles with Cl ions. Over time, Mn‐doped sensors demonstrate improved stability and sensing capabilities, while the response of pure CsPbBr_3_ sensors declines. Undoped mixed halides and CsPbCl_3_ sensors maintain a stable but low response, with limited differentiation between O_3_ levels. In contrast, Mn‐doped sensors preserve their sensing abilities, with the 50% v/v Mn‐doped sensor exhibiting the best response and stability. The room‐temperature‐operating sensors fabricated from Mn‐doped perovskites exhibit a reduced response in comparison to the advanced metal oxide sensors that require light irradiation.^[^ ^65^, ^66^, ^67^ ^]^ However, their ability to operate effectively and recover at room temperature is a significant advantage. Furthermore, when assessing stability, the manganese‐doped perovskite sensors demonstrate superior stability relative to the previously reported organic‐inorganic metal halide perovskite‐based sensors.^[^ ^27^ ^]^

These findings offer valuable insights into the gas detection capabilities of halide perovskites, showcasing the intricate relationship between metal ions, halides, and surface defects. By manipulating halide composition and manganese doping levels, sensor response and stability can be tailored, paving the way for affordable, sensitive, and energy‐efficient gas sensors with potential for selective detection. This study not only advances gas detection technologies but also provides vital information for enhancing the long‐term stability and reliability of perovskite materials across various applications, including photovoltaics and optoelectronics. The demonstration of tunable sensitivity in mixed halide perovskites, coupled with the enhanced performance achieved through doping, opens new avenues for designing bespoke sensing materials. The ability to control bandgap and defect concentrations through compositional engineering enables the optimization of sensor response to specific ozone concentrations or other target gases. Moreover, the insights gained into degradation pathways and long‐term stability are paramount for developing durable and reliable sensors for real‐world deployment.

## 4 Experimental Section

### Chemicals

The chemical precursors CsBr (99.999% trace metals basis), PbBr_2_ (≥ 98% trace metals basis), CsCl (≥99.5% trace metals basis), PbCl_2_ (99.999% trace metals basis), and MnCl_2_ (≥ 99% trace metals basis) were purchased from Sigma Aldrich and used without further treatment before the synthesis of the metal halide perovskite µCs. The solvents N, N‐Dimethylformamide (DMF, ≥99.8%), toluene (≥ 99.5%), and Dimethyl Sulfoxide (DMSO, ≥99.7%) were purchased from Honeywell, Sigma‐Aldrich and the Merck, respectively.

### Synthesis of CsPbBr_3_ µCs

Ligand‐free CsPbBr_3_ µCs were synthesized via a room temperature re‐precipitation‐based approach. A precursor solution was prepared by dissolving 0.4 mmol of CsBr and 0.4 mmol of PbBr_2_ in 10 ml of DMF under ambient conditions. Subsequently, 1 ml of this precursor solution was added into 2 ml of toluene and the color of the solution turned instantly into bright yellow, indicating the rapid nucleation and crystal formation. Following this, the mixture was sonicated for 30 min to achieve a uniform µCs size distribution.

### Synthesis of CsPbCl_3_ µCs

Similar to the CsPbBr_3_ µCs, CsPbCl_3_ µCs were formed by adding 1 ml of the stock solution into 2 ml of toluene, followed by a 30 min sonication. In this case, the precursor solution was prepared by dissolving 0.4 mmol of CsCl and 0.4 mmol of PbCl_2_ in 10 ml of DMSO.

### Synthesis of Undoped CsPbBr_3‐x_Cl_x_ µCs

CsPbBr_3‐x_Cl_x_ µCs were synthesized through a typical anion exchange reaction. Initially, a PbCl_2_ precursor solution was prepared by dissolving 0.6 mmol of PbCl_2_ in 5 ml DMF. Following this, four distinct mixtures were prepared by adding precise amounts of PbCl_2_ into the pre‐synthesized CsPbBr_3_ µCs with volume‐to‐volume ratio of 10%, 20%, 50% and 80%. To accelerate the anion exchange process, the µCs were placed in an ultrasonic bath for 30 min.

### Synthesis of Mn‐Doped CsPbBr_3‐x_Cl_x_ µCs

Mn‐doped CsPbBr_3‐x_Cl_x_ µCs were synthesized via a modified post‐synthetic halide exchange‐driven cation exchange strategy (HEDCE) at room temperature.^[^ ^37^ ^]^ A stock solution of MnCl_2_ precursor was synthesized by dissolving 0.6 mmol of MnCl_2_ powder into 5 ml of DMF. Subsequently, precise volumes of the stock MnCl_2_ solution were added into the pre‐formed of CsPbBr_3_ µCs and the mixture was sonicated for 30 min. The doping level was controlled by using the same volume‐to‐volume ratios as those employed for the undoped mixed halides µCs, which was 20%, 50%, and 80%.

### Characterization of the Materials

Scanning Electron Microscopy (SEM, JEOL 7000) incorporated with Energy Dispersive X‐Ray Spectrometer (EDS, INCA PentaFET‐x3) operating at 20 kV was employed to analyze the surface morphology and elemental composition of the fabricated microcrystals. The crystal structures were investigated by X‐Ray Diffraction (XRD, Bruker AXS D8 Advance copper anode diffractometer) over the 2θ collection range of 10° to 50° with scan rate of 0.02° s^−1^, using a monochromatic Cu Kα radiation source (λ = 1.54056 Å). SEM and XRD samples were prepared by drop‐casting the µCs on indium tin oxide (ITO) coated glass substrates and glass substrates, respectively. The relative concentration Mn/Pb in Mn‐doped mixed halide systems was estimated by Inductively Coupled Plasma Mass Spectroscopy (ICP‐MS, Perkin Elmer NexION 350D) which provides a doping level representative for the whole sample. The microcrystals were in powder form and were diluted in 3 mL of 2% HNO_3_ and heated at 95 °C overnight on heating blocks. The absorbance spectra of all the fabricated materials were measured by UV‐Visible spectroscopy (Perkin Elmer Lambda 950 UV/Vis/NIR) over the range of 360 nm to 650 nm after drop‐casting the microcrystals on glass substrates. The chemical compositions and electronic states of the perovskite‐based microcrystals, drop‐casted on ITO coated glass substrates, were determined by X‐Ray Photoelectron Spectroscopy (XPS, SPECS‐Germany, FlexMod) equipped with a PHOIBOS 100 1D‐DLD energy analyzer and an Al Kα monochromatic X‐Ray source (1486.7 eV) operated with 200 W and 15 kV. The recorded survey spectra were the following: C 1s, Pb 4f, Br 3d, Cs 3d, Cl 2p, Mn 2p, and O 1s which recorded at a pass energy of 30 eV. All the binding energies in XPS spectra were calibrated using the C 1s at 284.8 eV as a reference. The data analysis was performed with SpecsLab Prodigy and CasaXPS (Casa software Ltd, DEMO version). A Tougaard baseline was used in combination with a Gaussian‐Lorentzian function for the high‐resolution spectra deconvolution, which was executed by OriginPro 2016 software.

### Preparation of the Electrodes and Gas Sensing Experiments

The perovskite µCs were drop‐casted onto commercially available interdigitated platinum electrodes on glass substrate (IDEs, 5 microns lines and gaps/glass substrate, Metrohm) and were left to dry under vacuum.

The gas sensing measurements were conducted at room temperature, in a custom‐made gas sensing chamber, providing a controlled environment for the electrical assessment of the sensors. The gas sensing setup consists of certified gas suppliers connected with mass flow controllers, coupled with a stainless chamber which was initially evacuated down to 10^−3^ mbar. To evaluate the ozone sensing capability of all fabricated materials, a commercial ozone analyzer (Thermo Electron Corporation, Model 49i) was employed. The analyzer was used to supply and record accurately controlled ozone concentrations that were introduced in the chamber at a flow rate of 500 sccm (standard cm^3^ min^−1^) flow. Electrical current measurements over time were executed using an electrometer (Keithley 6517A) by applying a constant voltage. The values of the voltage ranged from 1 to 3.5 V, depending on the conductivity of each sensor. The sensing process was initiated by exposing the sensors to ozone gas for 150 s, followed by a 200 s recovery with synthetic air. All samples were exposed to different ozone concentrations ranging from 1567 down to 4 ppb. During the experimental procedure, the pressure in the chamber maintained constant to 700 mbar.

The performance of the sensors for each gas is assessed through their response (S), defined as:

(1) $$S=\frac{I_{\mathrm{gas}}}{I_{\mathrm{air}}}\;\mathrm{or}\;S=\frac{I_{\mathrm{air}}}{I_{\mathrm{gas}}}\;$$

where *I*_gas_ and *I*_air_ are the electrical current values after saturation in the presence of reducing/oxidizing gas and synthetic air, respectively.

The percentage response difference between the as prepared and aged µCs’ samples were calculated by the equation:

(2) $$\mathrm{Percentage}\;\mathrm{response}\;\mathrm{difference}=\frac{r_{\mathrm{aging}}-r_{\mathrm{as}-\mathrm{prepared}}}{\left[\frac{r_{\mathrm{aging}}+r_{\mathrm{as}-\mathrm{prepared}}}{2}\right]}\times 100$$

### Computational Methodology

The (001) oriented CsPbBr_3_ surfaces were simulated by five‐layer slab models, as suggested by W. Xing et.al., built with a 2 × 2 supercell of the bulk orthorhombic phase of CsPbBr_3_ with atom positions and lattice parameters taken from the experimental data of C.C. Stoumpos et al.^[^ ^42^, ^68^ ^]^ From this bulk orthorhombic structure, symmetric slabs consisting of alternating PbBr_2_ and CsBr layers were built, terminated by PbBr_2_ planes. The constructed slabs with lattice constants a = 11.6264 Å, b = 11.7351 Å, c = 32.6424 Å are finite in the z‐direction, separated by a vacuum gap of 20 Å, and periodic in x‐ and y‐ directions. Modified surfaces with Br and Pb vacancy were constructed by removing 1 Br and 1 Pb atom from the PbBr_2_ termination plane, respectively, while Mn‐doped CsPbBr_3_ surface was constructed by substituting 1 Pb atom with 1 Mn atom, and Cl‐doped by substituting 1 Br atom with 1 Cl atom, or all Br atoms from the uppermost layer of the simulated surface with Cl atoms. The (001) oriented CsPbCl_3_ surface presented in the Supporting Information section was constructed by using the bulk tetragonal structure with lattice constants obtained from the Materials Project database,^[^ ^69^ ^]^ while the corresponding defected surface with a Cl vacancy was constructed by removing 1 Cl atom from the PbCl_2_ termination plane.

DFT calculations were performed as implemented in the Vienna ab initio simulation package (VASP).^[^ ^70^, ^71^ ^]^ The generalized gradient approximation (GGA) with the Perdew‐Becke‐Erzenhof (PBE) parametrization for the exchange‐correlation (XC) was employed, while interactions of valence electrons with the remaining ions were modeled within the projector augmented wave (PAW) formalism.^[^ ^72^, ^73^ ^]^ An energy cut‐off of 400 eV for the plane wave expansion was used and van der Waals interactions were taken into account employing the DFT‐D3 scheme of Grimme.^[^ ^74^ ^]^ An energy convergence criterion of 10^−6^ eV was used for the relaxation of the electronic degrees of freedom and all structures were considered fully relaxed when the maximum force acting on each atom was less than 0.01 eV Å^−1^. For the Brillouin zone integrations, a 4 × 4 × 1 Γ‐centered k‐point mesh was selected and a Gaussian smearing of 0.1 eV was used. During structure optimization, the cell volume and shape were not allowed to change, the positions of the atoms in the bottom three layers of the slabs were held fixed, while the atoms in the top two layers and of the adsorbate were allowed to relax.^[^ ^75^ ^]^

The adsorption energies, ΔE_ads_, were calculated as  Δ*E*_ads_ = *E*_O2 + slab_  − *E*_slab_ − *E*_O2_, where *E*_O2 + slab_is the energy of the slab with the adsorbed O_2_ molecule, *E*_slab_ is the energy of the slab, and *E*_*O*2_ is the spin‐polarized energy of the O_2_ molecule in vacuum.

Charge density difference plots were calculated as the difference of the electron density of the slab + O_2_ complex system minus the sum of the isolated slab and O_2_ within the conformation of the complex, and were plotted using VESTA software on a 0.005 (e bohr^−3^) isosurface.^[^ ^76^ ^]^

## Conflict of Interest

The authors declare no conflict of interest.

## Supporting information



Supporting Information

## Data Availability

The data that support the findings of this study are available from the corresponding author upon reasonable request.
