# Supplementary material for: Toward the Optimization of a Perovskite‐Based Room Temperature Ozone Sensor: A Multifaceted Approach in Pursuit of Sensitivity, Stability, and Understanding of Mechanism
Source: Small. 2025 Jan 9;21(6):2404430. doi: 10.1002/smll.202404430 (PMC11817909; doi:10.1002/smll.202404430)
Supplement: Supplementary file 1 — Supporting Information [file SMLL-21-2404430-s001.docx]

Supporting Information

**Towards the optimization of a perovskite-based room temperature ozone sensor: A multifaceted approach in pursuit of sensitivity, stability, and understanding of mechanism**

Aikaterini Argyrou, Rafaela Maria Giappa, Emmanouil Gagaoudakis, Vassilios Binas, Ioannis Remediakis, Konstantinos Brintakis*, Athanasia Kostopoulou*, Emmanuel Stratakis*

**S1. Structural and sensing properties of the metal halide perovskite microcrystals**

**
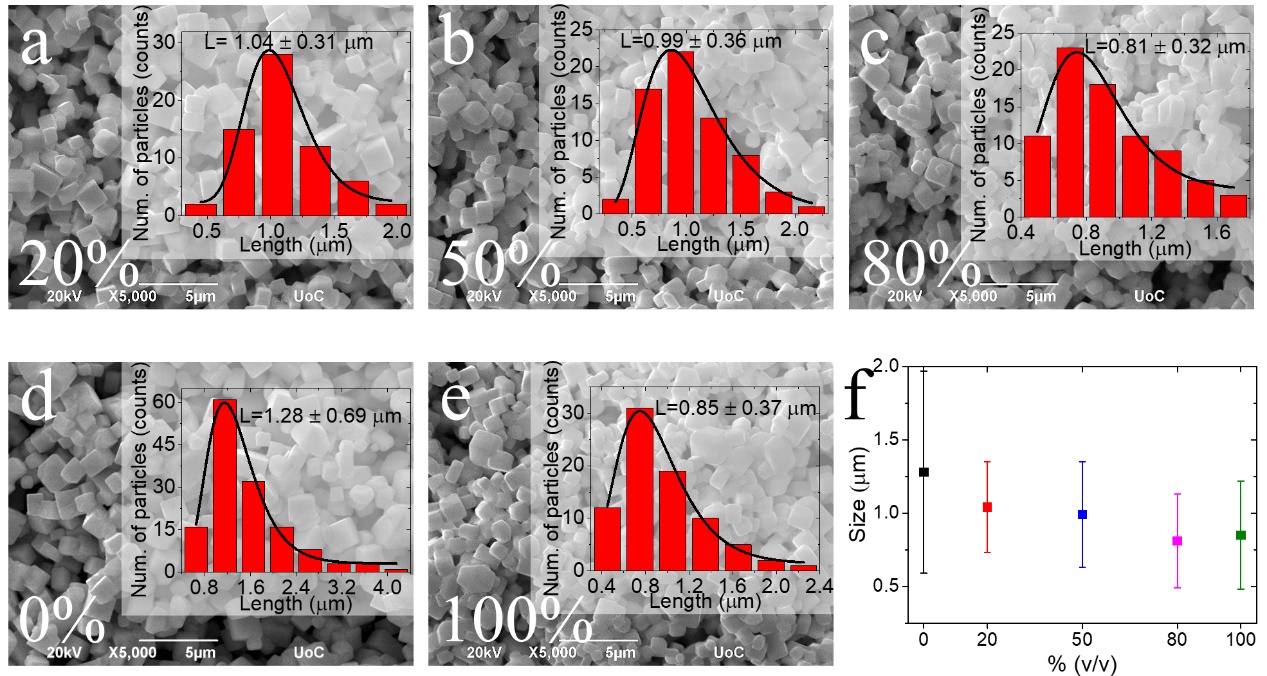
**

**Figure S1.** SEM images of undoped mixed halide perovskites with volume-to-volume ratios of a) 20%, b) 50%, c) 80% and of the reference samples d) CsPbBr_3,_ 0%, e) CsPbCl_3_, 100% v/v. The insets show the size distribution diagrams and the average μC size for each sample. f) Average μCs size evolution by tuning the volume-to-volume ratio.

**
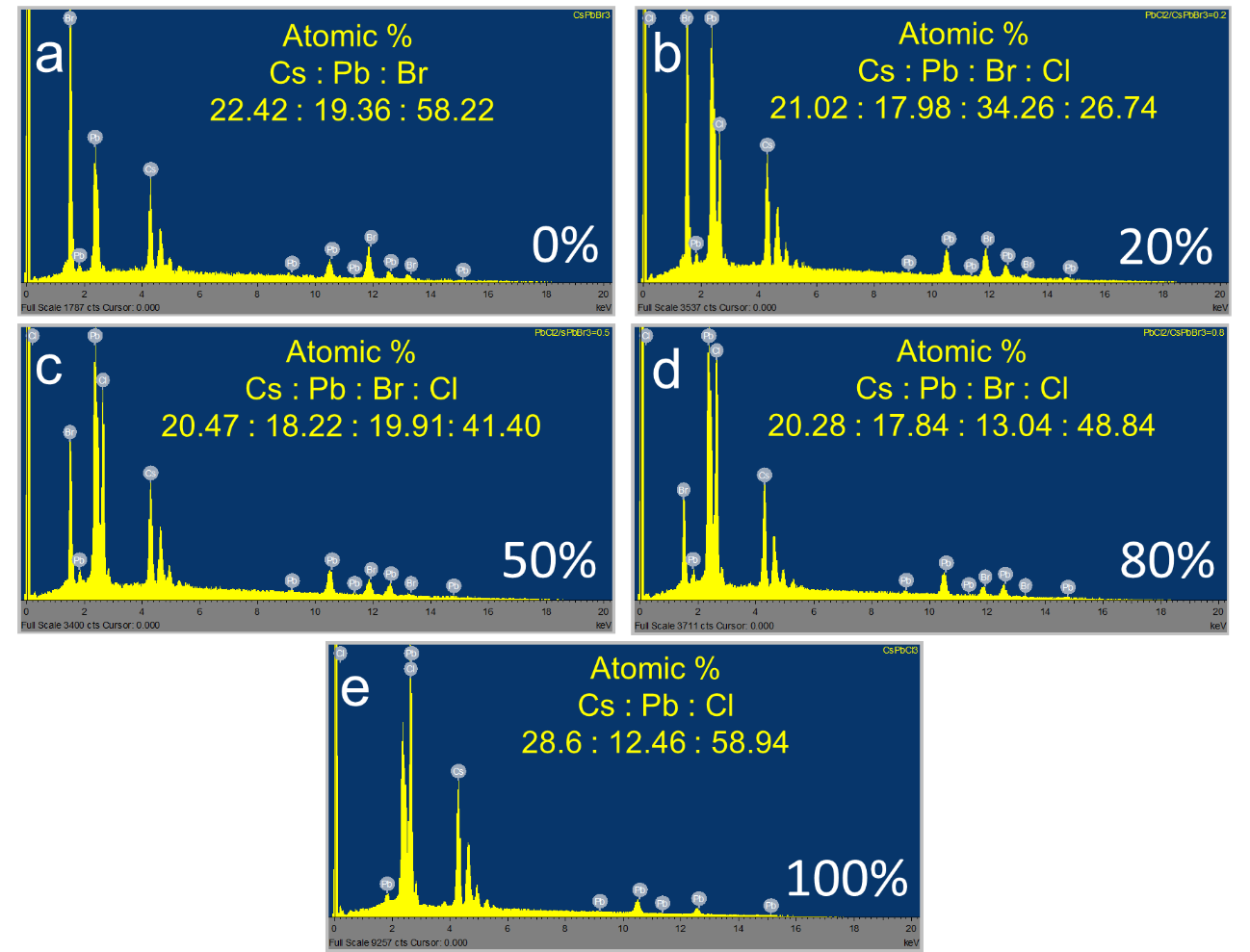
**

**Figure S2.** EDS spectra of the undoped CsPbBr_3-x_Cl_x_ perovskite μCs synthesized by varying the volume-to volume-ratio from 0% to 100%.

**Table S1.** Chemical composition and Cl/Br ratio of the undoped CsPbBr_3-x_Cl_x_ μCs as derived by EDS and XPS.

| % v/v | Chemical composition | Cl/Br  by EDS | Cl/Br  By XPS |
| --- | --- | --- | --- |
| 20 | CsPbBr_1.4_Cl_1.6_ | 0.78 | 1.06 |
| 50 | CsPbBr_1_Cl_2_ | 2.08 | 1.63 |
| 80 | CsPbBr_0.08_Cl_2.2_ | 3.74 | 2.79 |
| 0 | CsPbBr_3_ |  |  |
| 100 | CsPbCl_3_ |  |  |

**Figure S3.** XPS survey spectrum of CsPbBr_3_ μCs.

**Figure S4.** XRD patterns of 0% v/v CsPbBr_3_ (black) and 80% v/v undoped CsPbBr_3-x_Cl_x_ (red) μCs.

**
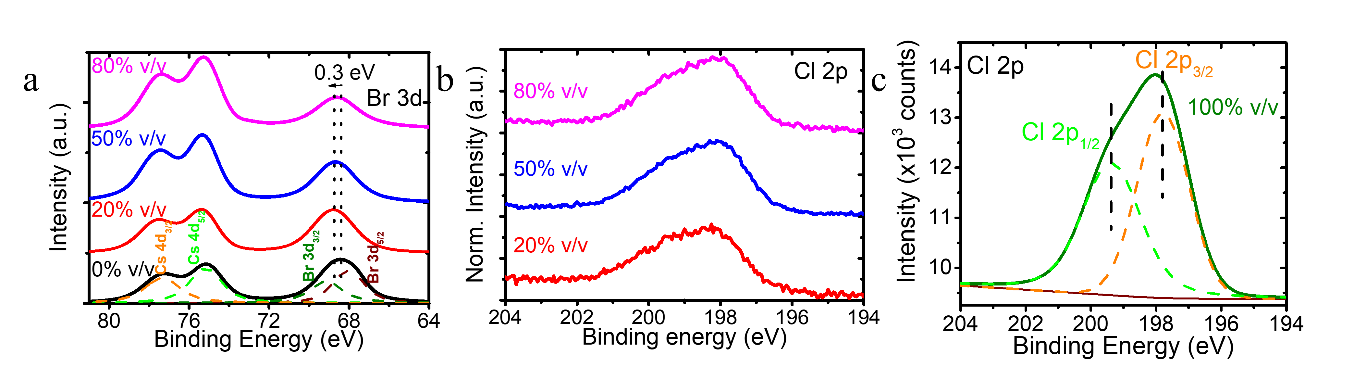
Figure S5.** High resolution XPS spectra of a) Br 3d, b-c) Cl 2p of 0% v/v CsPbBr_3_ (black curve), 20% v/v undoped (red curves), 50% v/v undoped (blue curves), 80% v/v undoped (magenta curves) CsPbBr_3-x_Cl_x_ μCs and 100% v/v CsPbCl_3_ (green curve). A thorough deconvolution of Br 3d and Cl 2p peaks of CsPbBr_3_ and CsPbCl_3_ respectively, revealed the typical d_5/2_/d_3/2_ and p_3/2_/p_1/2_ doublets at ~68.0 eV/~69.1 eV and ~197.8 eV/~199.4 eV for both peaks, which are associated with Pb-Br and Pb-Cl bonds (c).


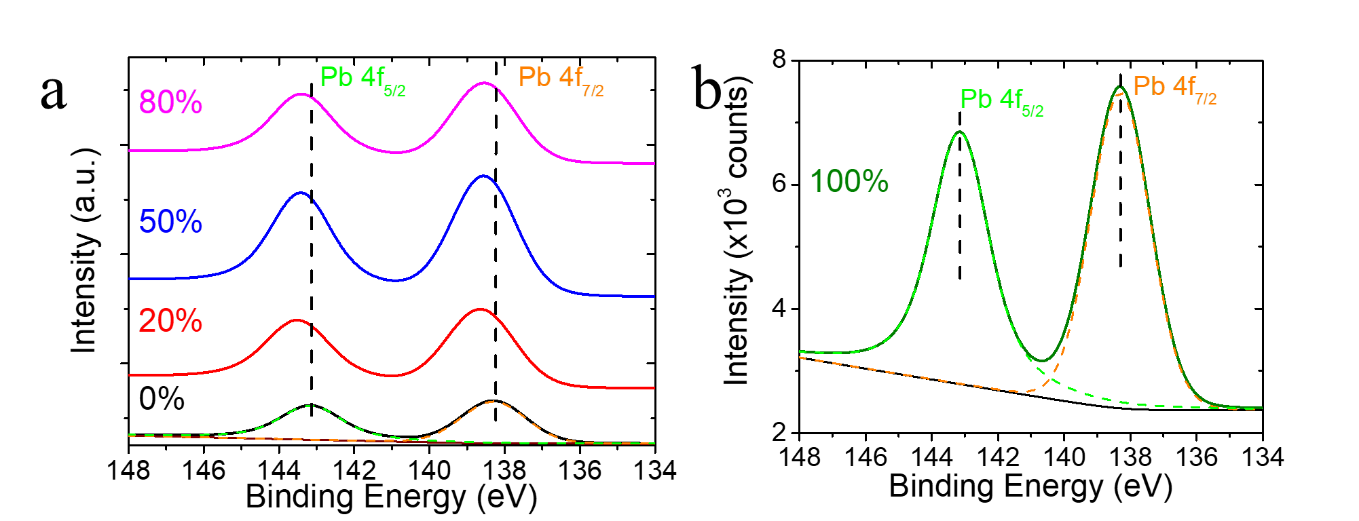
**Figure S6.** High-resolution XPS spectra of Pb 4f of a) undoped CsPbBr_3-x_Cl_x_ μCs (20 ,50, 80% v/v) and reference sample, 0% v/v CsPbBr_3_ μCs and b) the reference sample, 100% v/v CsPbCl_3_ μCs.


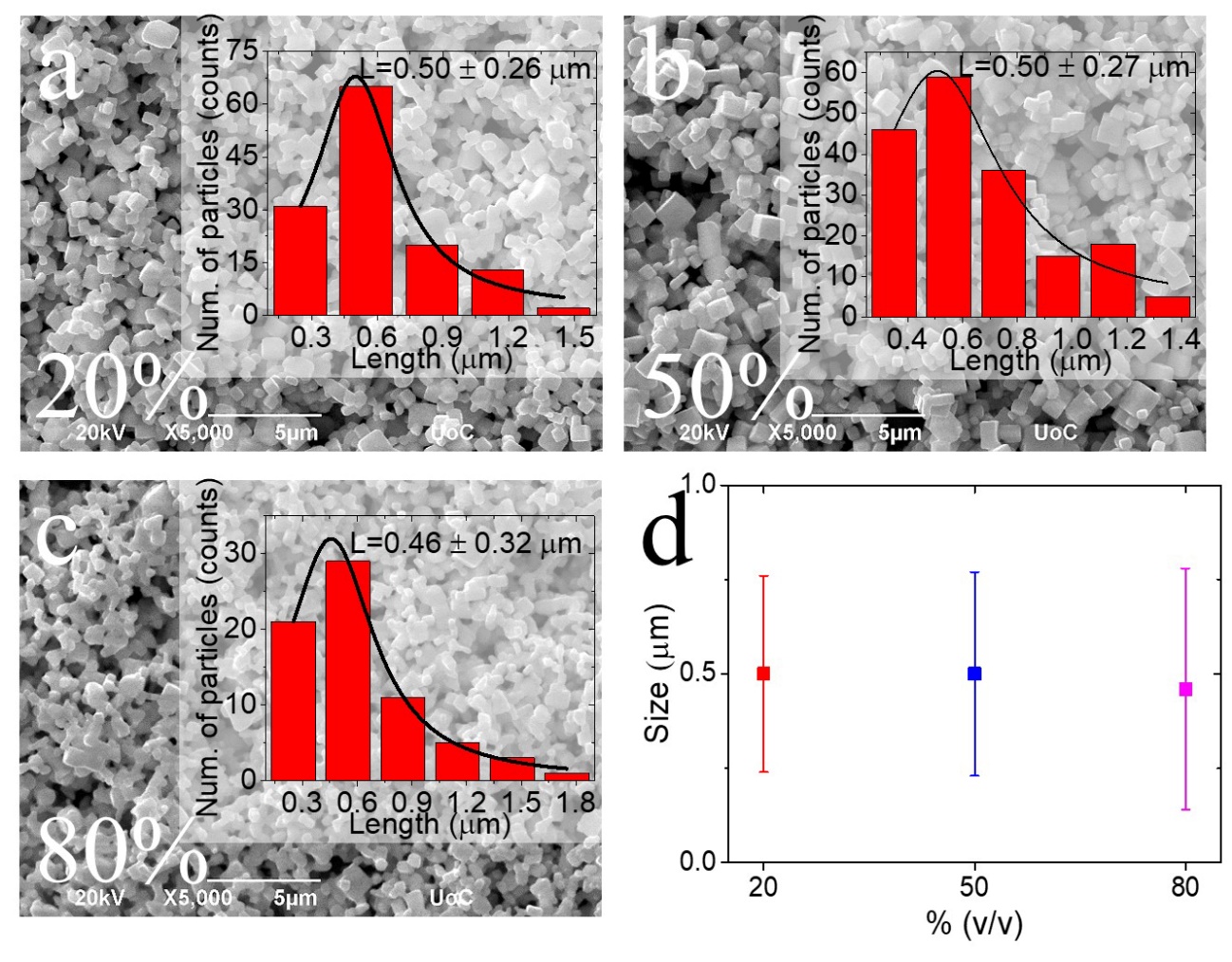


**Figure S7.** SEM images of Mn-doped CsPbBr_3-x_Cl_x_ μCs with volume-to-volume ratio of a) 20%, b) 50% and c) 80% v/v. The insets showed the size distribution diagrams and the average μC size for each sample. d) Average μCs’ size evolution by tuning the volume-to-volume ratio.


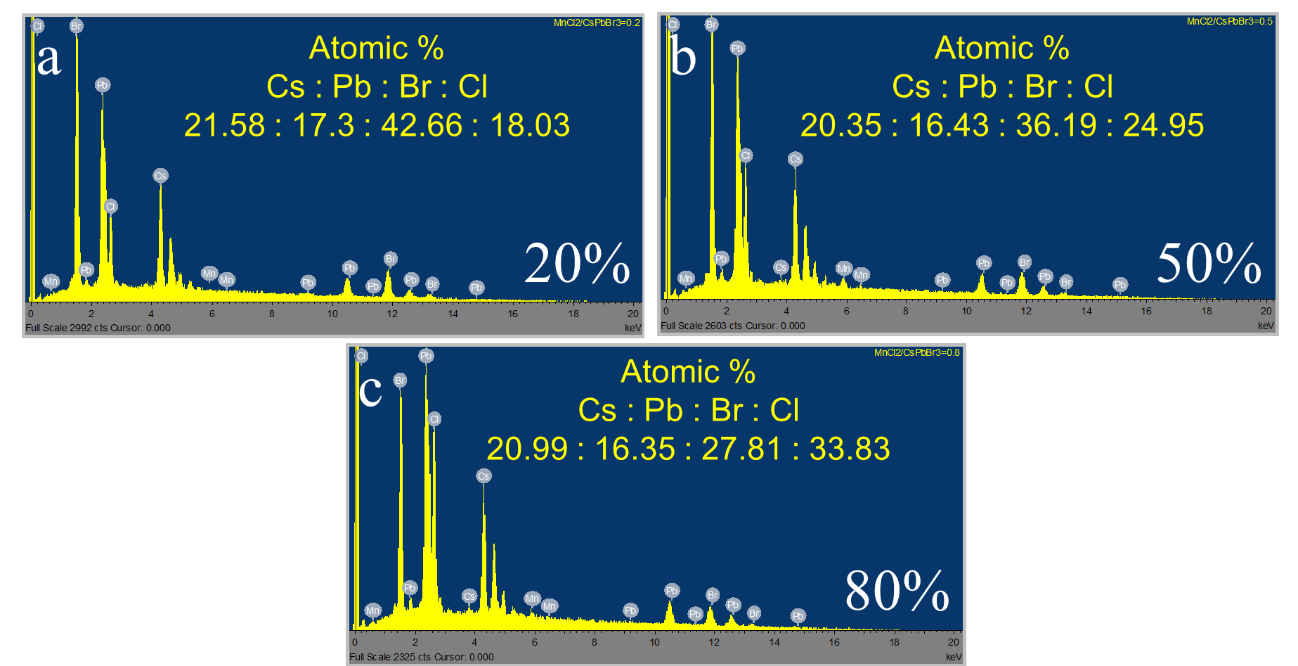
**Figure S8.** EDS spectra of Mn-doped CsPbBr_3-x_Cl_x_ μCs synthesized with a) 20%, b) 50% and c) 80% v/v.

**Table S2.** Chemical composition, Cl/Br ratio and Mn/Pb of the Mn-doped CsPbBr_3-x_Cl_x_ μCs as derived by EDS, XPS and ICP-MS.

| % v/v | Cl/Br  by EDS | Cl/Br  by XPS | Mn/Pb  by ICPMS |
| --- | --- | --- | --- |
| 20 | 0.59 | 0.42 | 0.06 |
| 50 | 1.32 | 0.95 | 0.09 |
| 80 | 1.75 | 1.46 | 0.14 |


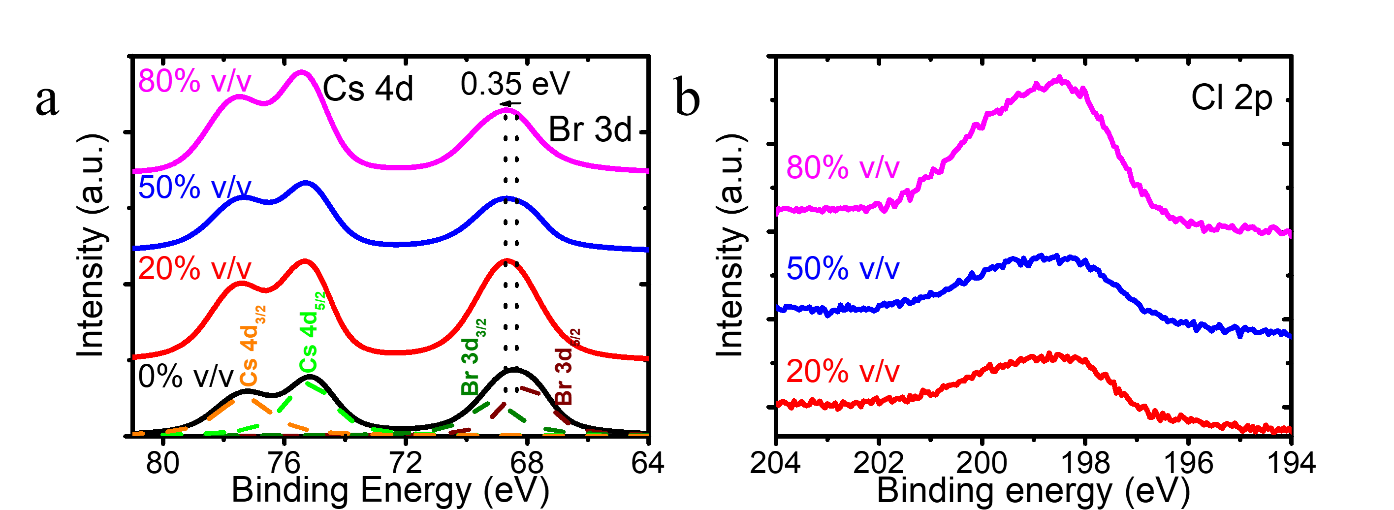


**Figure S9.** High-resolution XPS spectra of a) Br 3d and b) Cl 2p for the 0% v/v CsPbBr_3_ (black curve) and Mn-doped CsPbBr_3-x_Cl_x_ μCs with varying halide ratios.

**Figure S10.** High-resolution XPS spectra of Pb 4f for Mn-doped CsPbBr_3-x_Cl_x_ μCs with varying halide ratios.


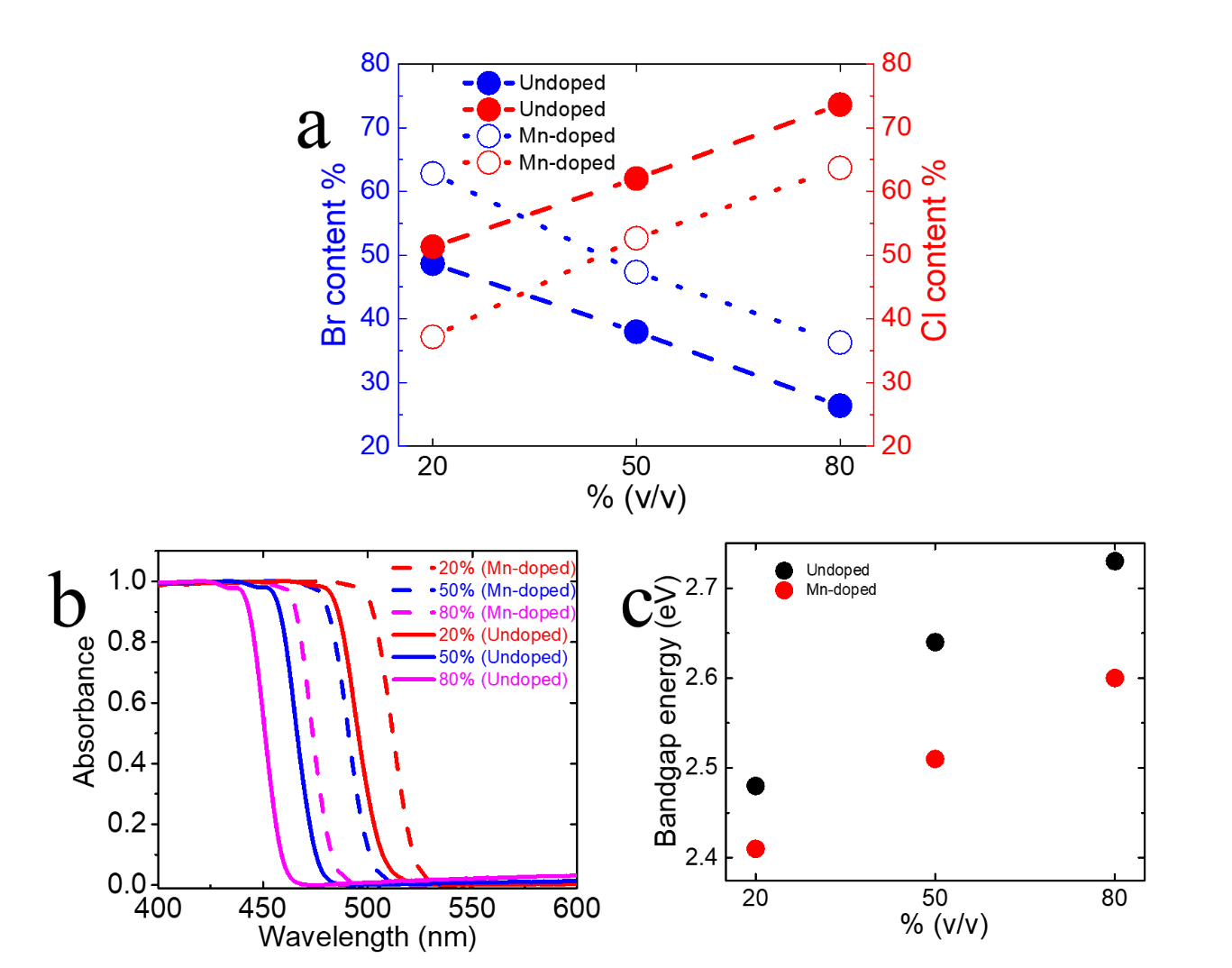


**Figure S11.** a) Halide content of undoped and Mn-doped CsPbBr_3-x_Cl_x_ μCs as calculated by XPS survey scans. b) Absorbance spectra of undoped (compact lines) and Mn-doped (dashed lines) CsPbBr_3-x_Cl_x_ μCs. c) Calculated bandgap energy of undoped (black circle) and Mn-doped (red circle) CsPbBr_3-x_Cl_x_ μCs.

**Figure S12.** XPS survey spectra for the Mn-doped CsPbBr_3-x_Cl_x_ μCs by varying the v/v ratio.

**Figure S13.** O_3_ sensing performance of the 10% v/v undoped perovskite-based sensor.

**Figure S14.** XPS survey spectra from bottom to top of 20% v/v (red line), 50% v/v (blue line) and 80% v/v (magenta line) undoped mixed halide μCs.

**S2. DFT calculations**

Modelling sensing based on first-principles calculations presents inherent challenges. While real-world sensing materials exhibit diverse morphologies and are often covered with various adsorbates at ambient conditions, DFT simulations generally assume idealized scenarios In the vast majority of such simulations, an ideal surface is assumed with only one kind of adsorbates, and adsorbates are arranged in a perfect periodic overlayer. These conditions are only met under ultra-high vacuum experiments at very low temperatures with large single-crystal samples. This discrepancy, known as the "pressure gap" in surface chemistry , highlights the difference between experimental conditions and simulations.^43^ Despite this gap, DFT simulations remain a valuable tool for identifying trends in surface reactivity. Experimental findings frequently confirm DFT predictions regarding the relative strength of molecular bindings, even though absolute binding energies may not align precisely.

***S2.1 Adsorption energies***

To construct a comprehensive understanding of the sensing mechanism, our investigation extended to the following model systems: (f) CsPbBr_3_ with Cl doping, where all eight surface Br atoms of the outmost surface layer are substituted by Cl atoms, (g) tetragonal CsPbCl_3_, and (h) tetragonal CsPbCl_3_ with one Cl vacancy site in the outmost surface layer.

**Table S3.** Adsorption energies of O_2_ adsorbed on the Cl-doped (001) surface of CsPbBr_3_ and on the defect-free and defected with Cl-vacancy (001) surface of CsPbCl_3_

| Model surface | ΔΕ_ads_  (eV) |
| --- | --- |
| 1 Cl-doped CsPbBr_3_ | 0.73 |
| 8 Cl-doped CsPbBr_3_ | 0.75 |
| Defect-free CsPbCl_3_ | 0.76 |
| CsPbCl_3_ with Cl-vacancy | -1.87 |

**
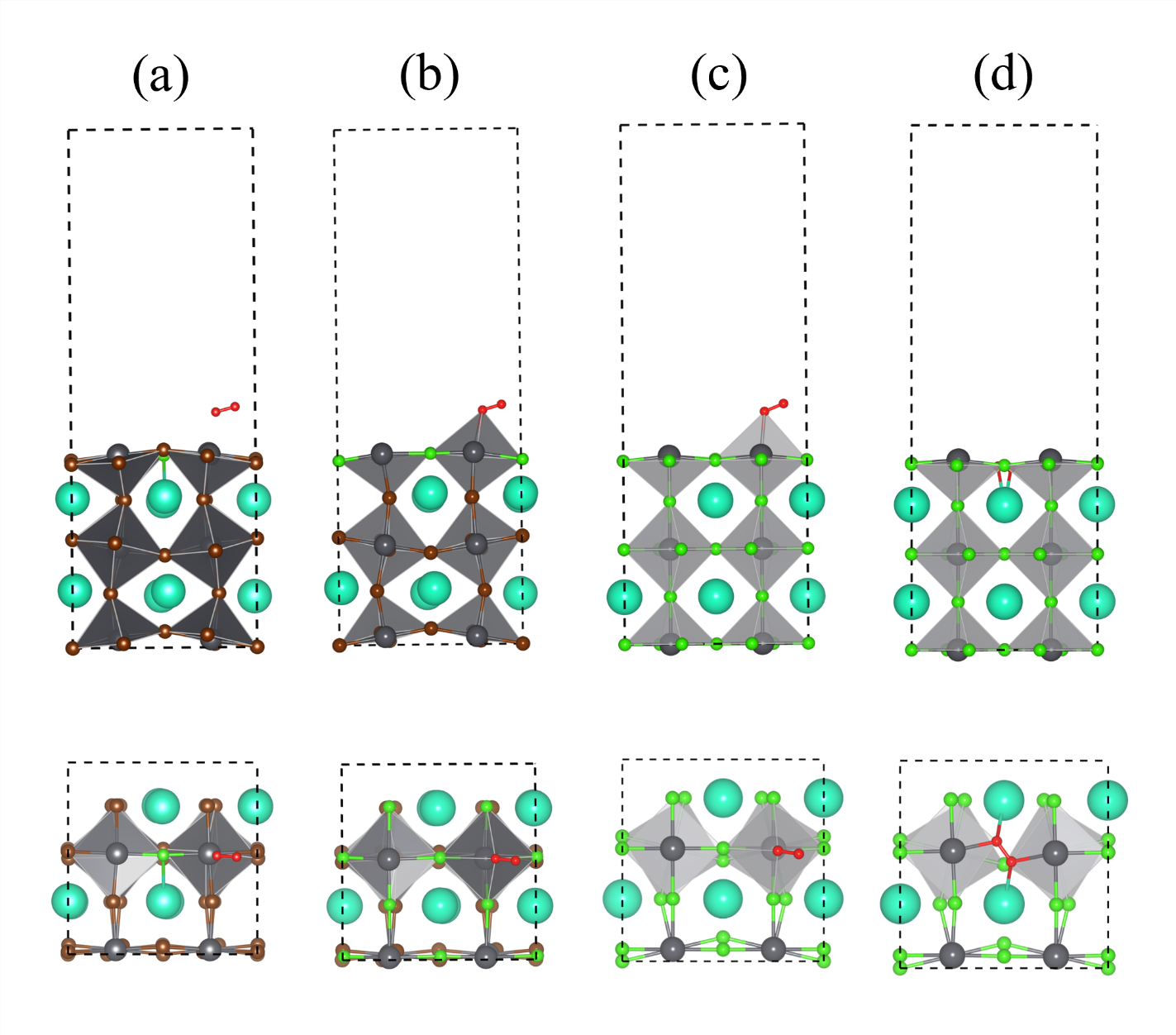
**

**Figure S15.** DFT-optimized configurations of the (001) oriented model surfaces; a) 1Cl-doped and b) 8Cl-doped CsPbBr_3_ model surface, viewed along b (upper line) and c (bottom line) axis. c) Defect-free CsPbCl_3_ and d) with Cl-vacancy model surface. Cs, Pb, Br, Cl, and O atoms are illustrated as cyan, gray, brown, green, and red atoms, respectively. Dashed lines indicate the periodic boundaries of the simulation supercell.

***S2.2 Electronic Properties***


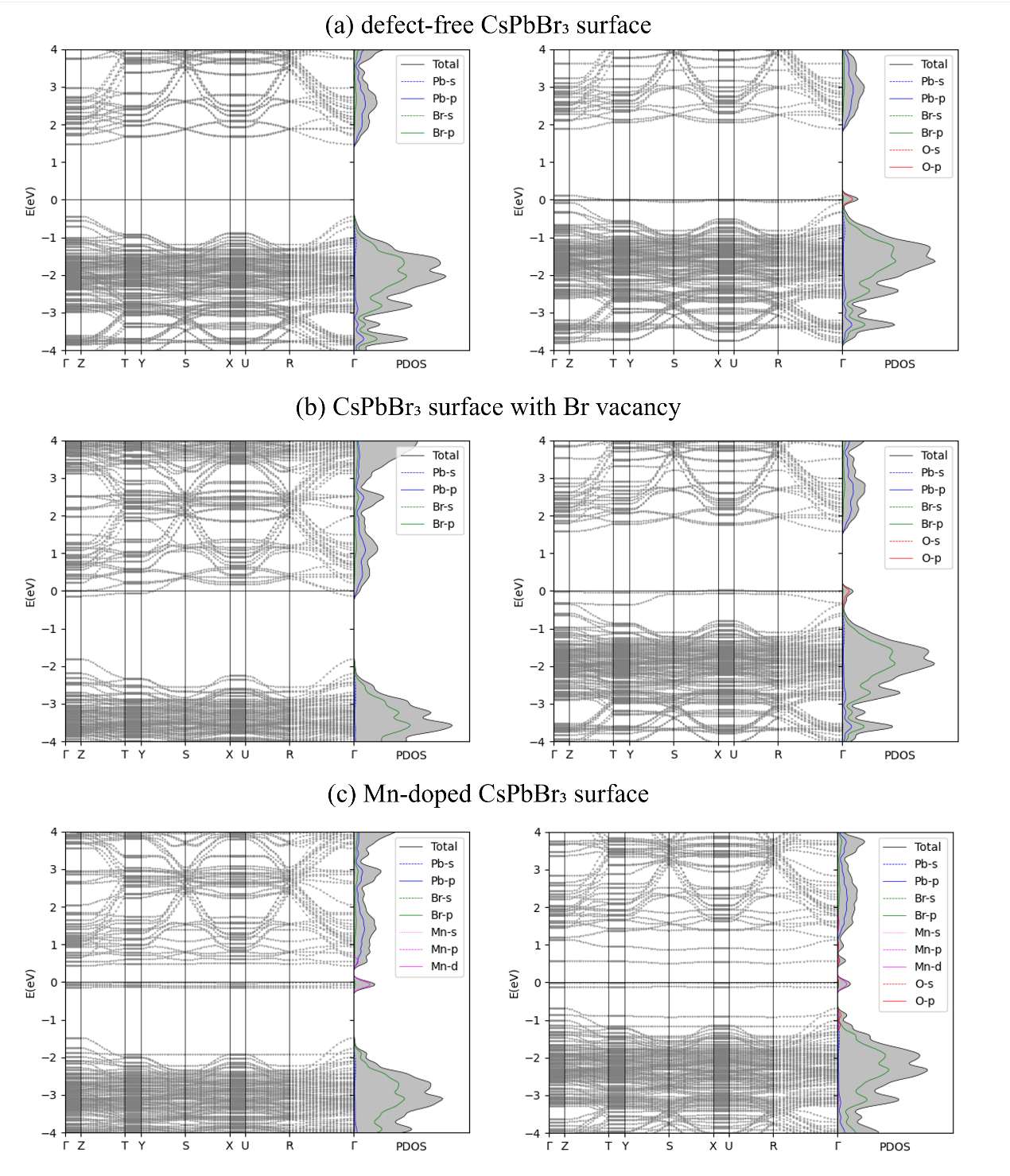


**Figure S16.** Band structures (BS) and partial density of states (PDOS) of the (001) oriented CsPbBr_3_ model surfaces without a) defects, b) with Br vacancy, and c) Mn-doped. For each system, the left panel corresponds to the model surfaces without oxygen, while the right panel refers to the surfaces with O_2_ adsorbed.

As it can be seen in Figure S16 (a), the valence band of defect-free surface is mainly occupied by the p orbitals of Br atoms, while the conduction band derives its contributions from the p orbitals of Pb atoms. When molecular oxygen is adsorbed on the defect-free surface, its p orbitals introduce new shallow acceptor states at the top of the valence band. Comparing the defect-free surface to the one with a Br vacancy (Figure S16b), new shallow states that provide electrons emerge at the bottom of the conduction band once a Br vacancy is introduced. However, upon oxygen adsorption on the Br vacancy, these shallow donor states are withdrawn, and new shallow acceptor states emerge at the top of the valence band, deriving their contributions from the p orbitals of oxygen atoms.

A rather interesting case emerges with the introduction of Mn to the lattice (Figure S16c) and was highlighted in the main text. Here, not only new donor states are observed in the conduction band, but also localized states from the d orbitals of Mn appear within the bandgap. Upon adsorption of oxygen on our Mn-doped model surface, the shallow donor states are quite withdrawn, and new acceptor states appear at the top of the valence band. The d-states of Mn remain localized at the same relative position within the gap, while the oxygen states exhibit a non-negligible density located both at the valence band and close to the conduction band minimum.

The band structure is altered also in the case of a Pb vacancy (Figure S17a) compared to the defect free case, with both the conduction and valence band appearing higher in energy. Oxygen adsorption in the Pb-vacancy introduces shallow acceptor states at the top of the valence band, which derive their contributions from the p orbitals of O and Br atoms.

For the halide substitution case, where one surface Br atom is substituted by one Cl atom (Figure S17b), we observe minor changes in the electronic structure. All the qualitative BS and PDOS characteristics remain similar to the defect-free case, as Cl states are located deep within the BS, far away from the CBM and VBM. When more Br atoms are substituted with Cl (Figure S16c), the contribution of Cl states in the VB becomes more significant, up to the case of CsPbCl_3_ (Figure S18a and b). Interestingly, the Fermi level shows a tendency to shift towards that of an n-doped system (Figure S19-22).

**
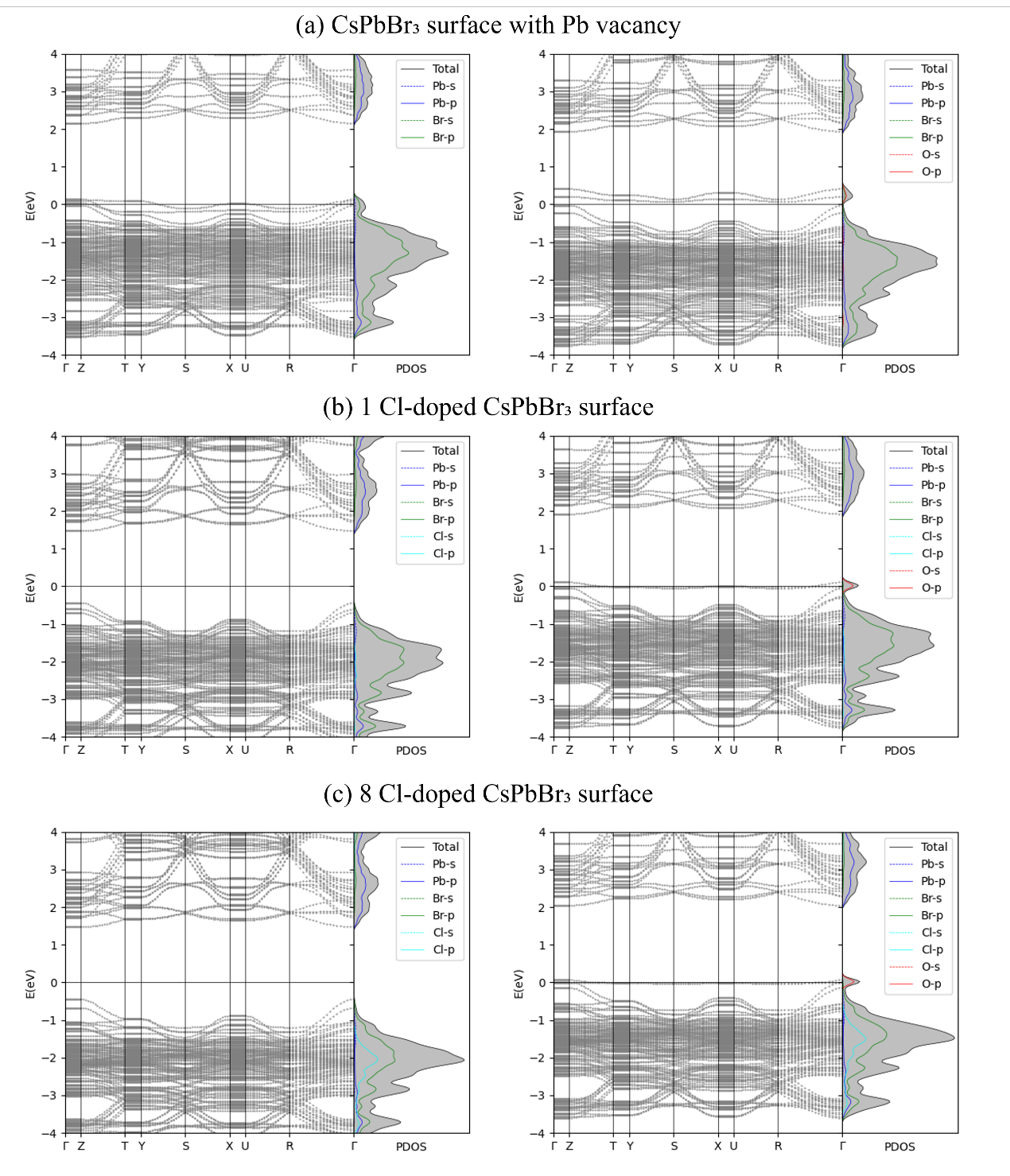
Figure S17.** Band structures (BS) and partial density of states (PDOS) of the (001) oriented CsPbBr_3_ model surfaces; with a) Pb vacancy, b)1Cl-doped and c) 8Cl-doped. For each system, the left panel corresponds to the model surfaces without oxygen, while the right panel refers to the surfaces with O_2_ adsorbed.


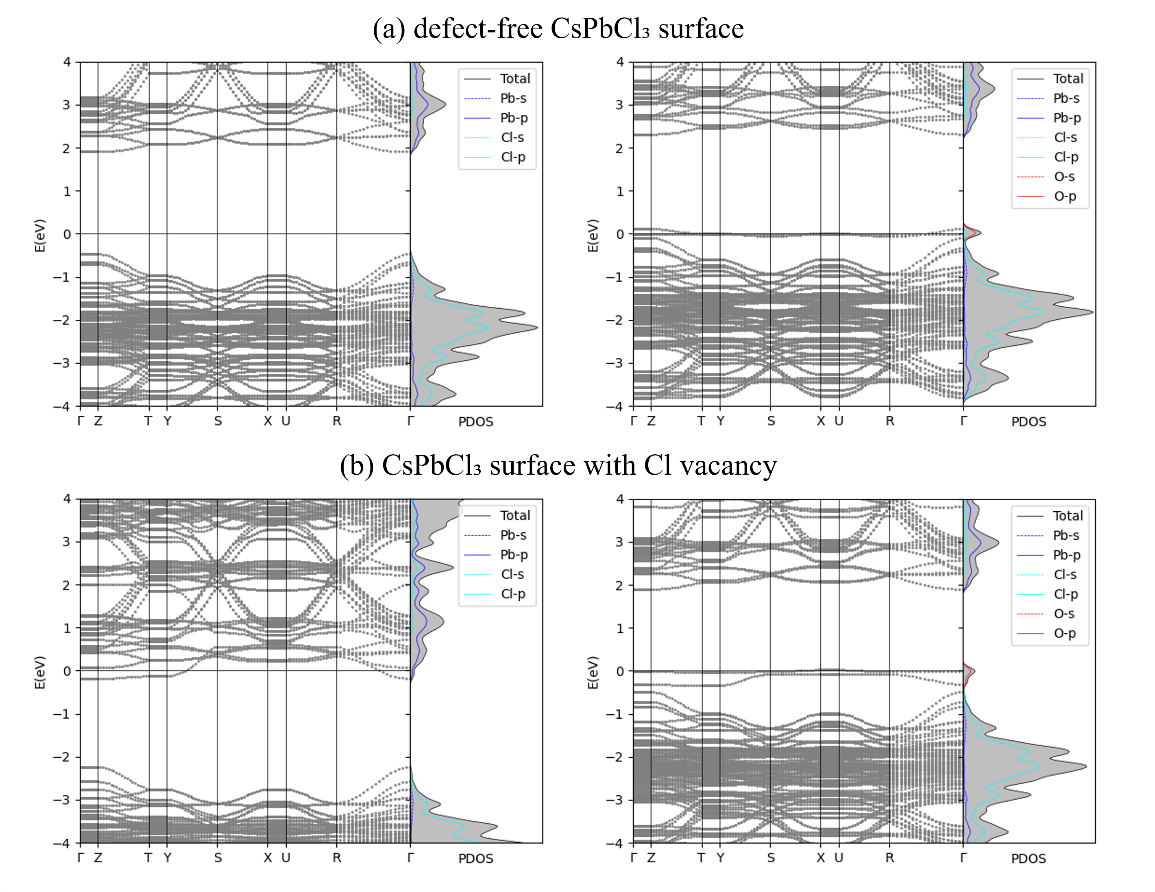


**Figure S18.** Band structures (BS) and partial density of states (PDOS) a) of the defect-free (001) oriented CsPbCl_3_ model surface and b) with Cl-vacancy. For each system, the left panel corresponds to the model surfaces without oxygen, while the right panel refers to the surfaces with O_2_ adsorbed.

***S2.3 Fermi shift***

In order to have a proper indication for the doping type of our model systems, we calculate the relative difference in their Fermi levels. Since DFT is well-known for accurately characterizing the DOS peaks below the Fermi level, we used the lowest energy peaks in the range -28 to -18 eV, which are identical in all systems (Figure S18). These peaks were found to have contributions only from the s orbitals of the Cs atoms in the case of the surfaces without O_2_ adsorbed and from Cs-s and O-s orbitals upon adsorption of molecular oxygen; orbitals that do not contribute to bond formation. Using the peaks shown in Figure S18a and the peaks from -22 to -19 eV shown in Figure S18b we shift the peaks of all systems (colored lines) towards the peak of the defect-free slab (denoted in black), a process demonstrated in Figure S19.

Indicatively, upon adsorption of molecular oxygen in the defect-free CsPbBr_3_ model surface (Figure S20a), states from the p-orbitals of oxygen emerge and the fermi level sinks 0.4 eV, suggesting a p-type behavior. Once a Br vacancy is introduced (Figure S20b), the fermi level rises 1.6 eV and “enters” the VB, indicating an n-type behavior. Once oxygen fills the Br vacancies, the n-type behavior diminishes as holes are provided by oxygen to the system, switching to a p-type behavior. In the case of oxygen adsorption on the Cl substituted CsPbBr_3_ model surface (Figure S21b and c), when more Cl atoms are substituting the Br atoms of the uppermost layer of the surface, the Fermi level shows a tendency to move towards that of an n-doped system.

**
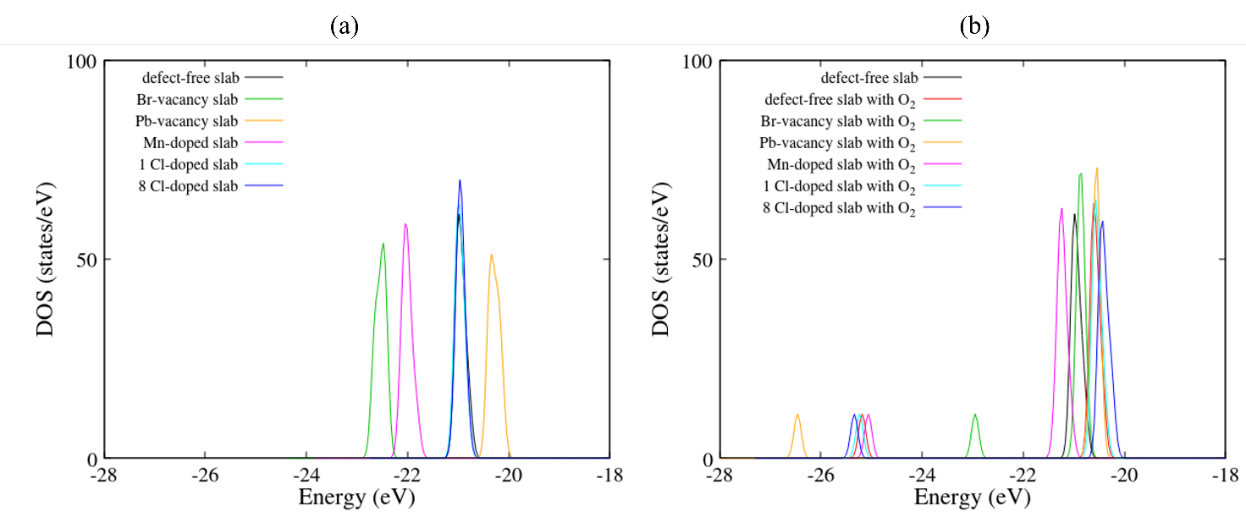
**

**Figure S19.** The lowest energy peaks of the DOS of all model CsPbBr_3_ surfaces without O_2_ adsorbed in (a) and upon adsorption of O_2_ in (b).


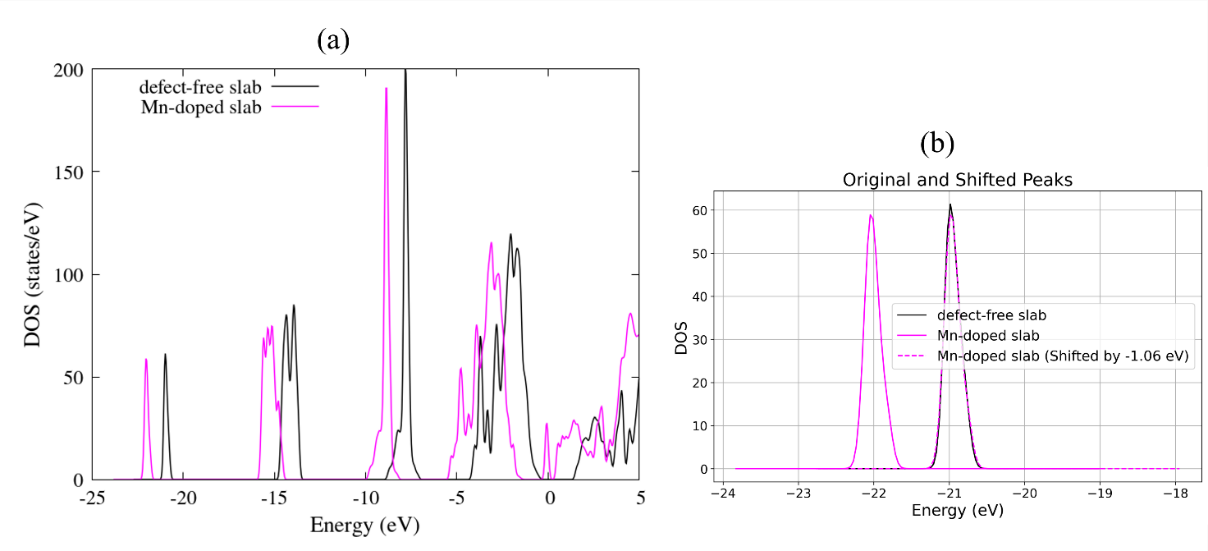


**Figure S20.** a) Total DOS of the defect-free and the Mn-doped CsPbBr_3_ slab in black and magenta, respectively. b) The lowest energy identical peaks (denoted in (a)) used to calculate the shift of the Fermi level.

**
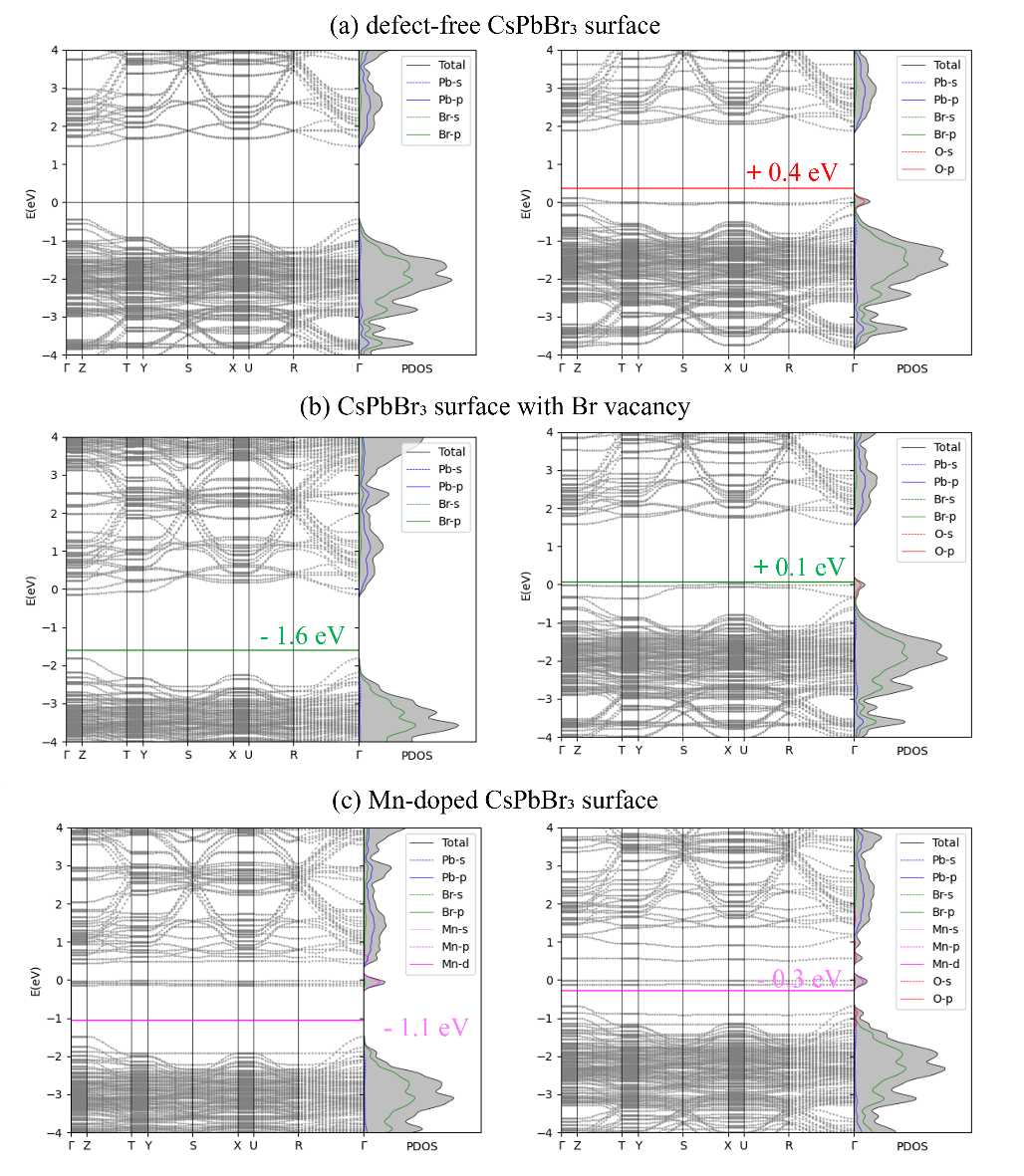
**

**Figure S21.** Band structures (BS) and partial density of states (PDOS) of the (001) oriented CsPbBr_3_  model surfaces a) without defects, b) with Br vacancy, and c) Mn-doped. For each system, the left panel corresponds to the model surfaces without oxygen, while the right panel refers to the surfaces with O_2_ adsorbed. Colored lines represent the fermi level shift of the defect-free system in each case.


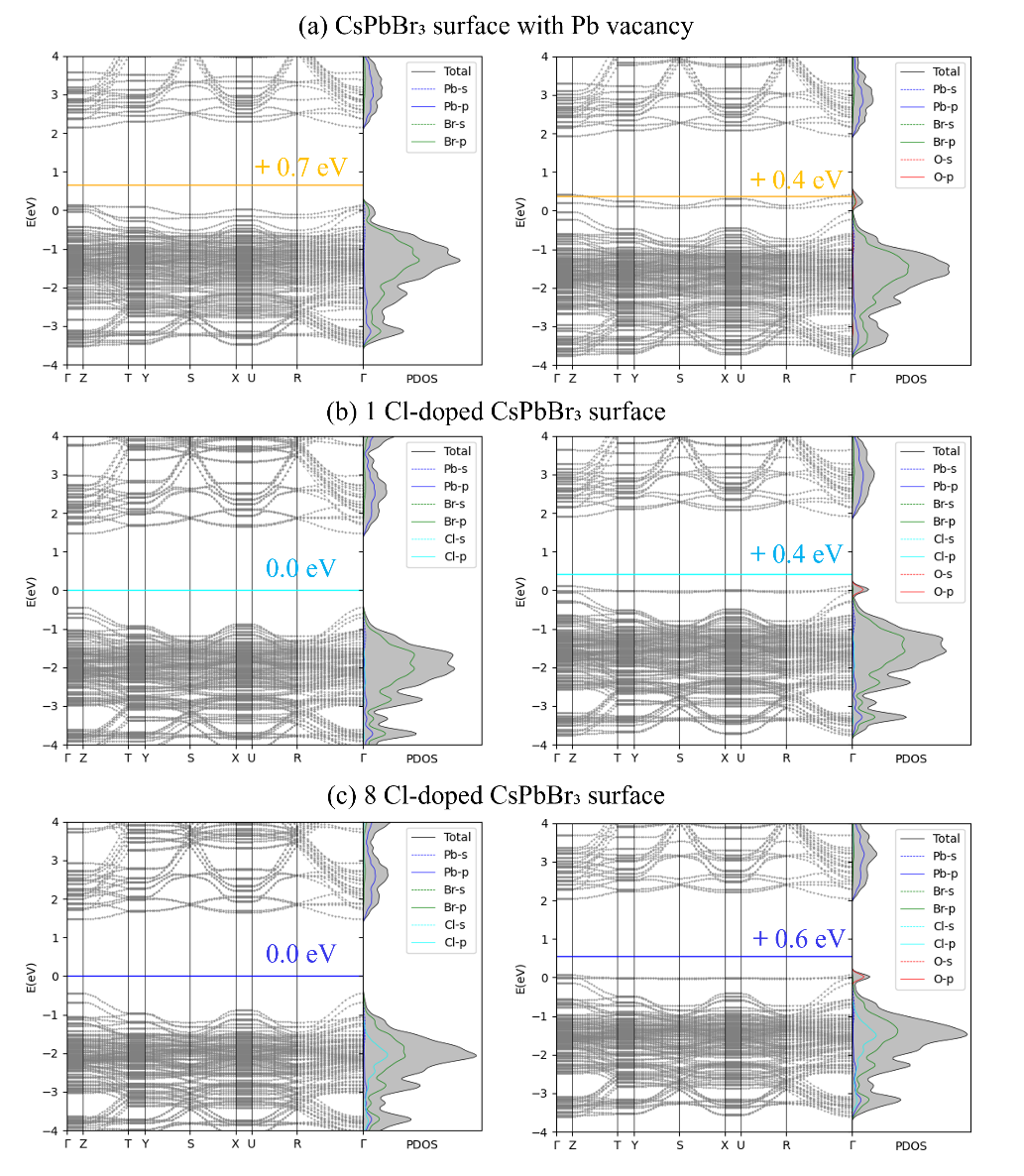


**Figure S22.** Band structures (BS) and partial density of states (PDOS) of the (001) oriented CsPbBr_3_  model surface with a) Pb vacancy, b) 1Cl-doped, and c) 8Cl-doped. For each system, the left panel corresponds to the model surfaces without oxygen, while the right panel refers to the surfaces with O_2_ adsorbed. Colored lines represent the fermi level shift of the defect-free system in each case.

***S3. Aging study and sensing properties***


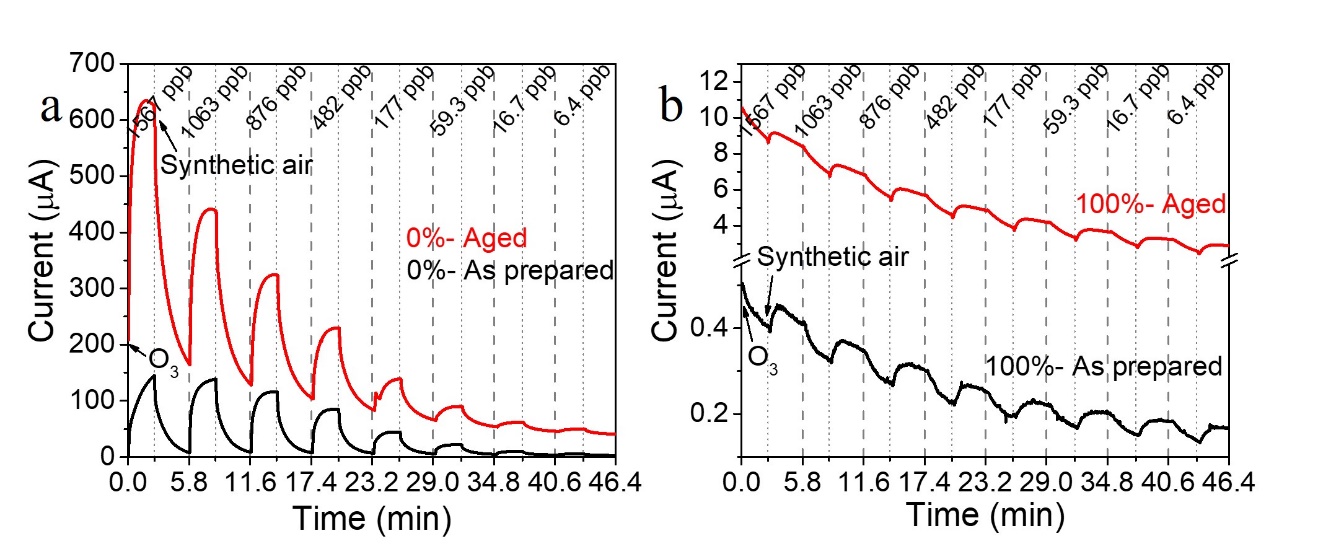
**Figure S23.** O_3_ sensing performance of the as-prepared (black) and aged for a month (red) reference samples, a) 0% v/v CsPbBr_3_ and b) 100% v/v CsPbCl_3_ μCs.

**
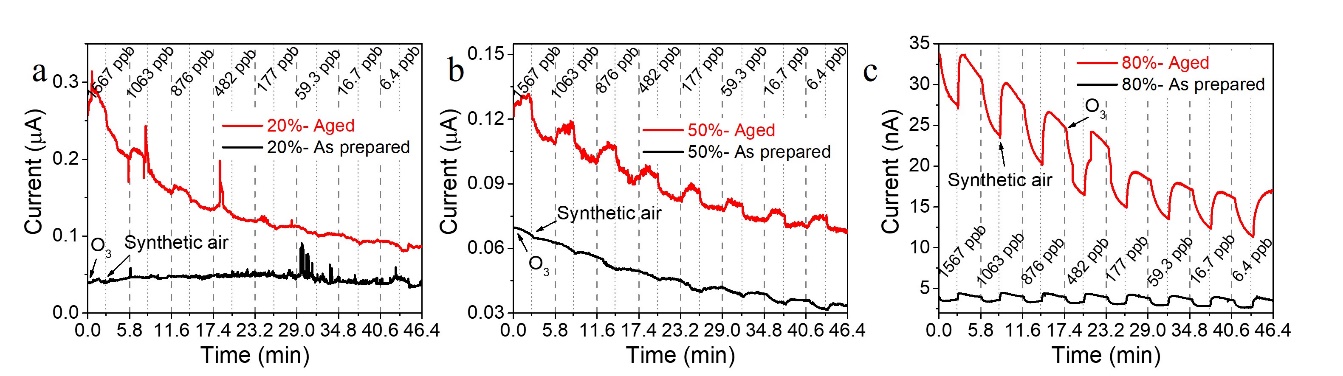
Figure S24.** O_3_ sensing performance of a) 20%, b) 50% and c) 80% v/v, as prepared (black) and aged (red) undoped perovskite-based sensors.

**
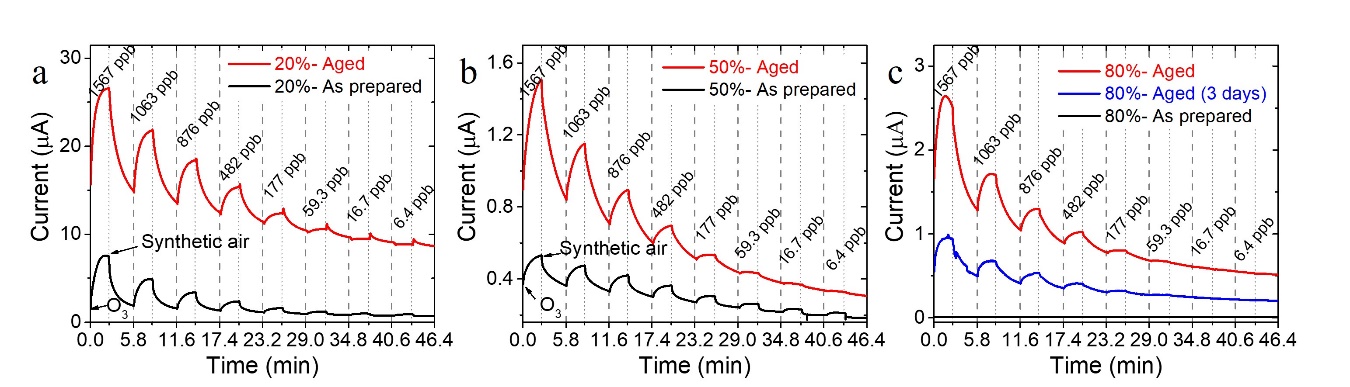
Figure S25.** O_3_ sensing performance of a) 20%, b) 50% and c) 80% v/v, as prepared (black) and aged (red) Mn-doped perovskite-based sensors.

**
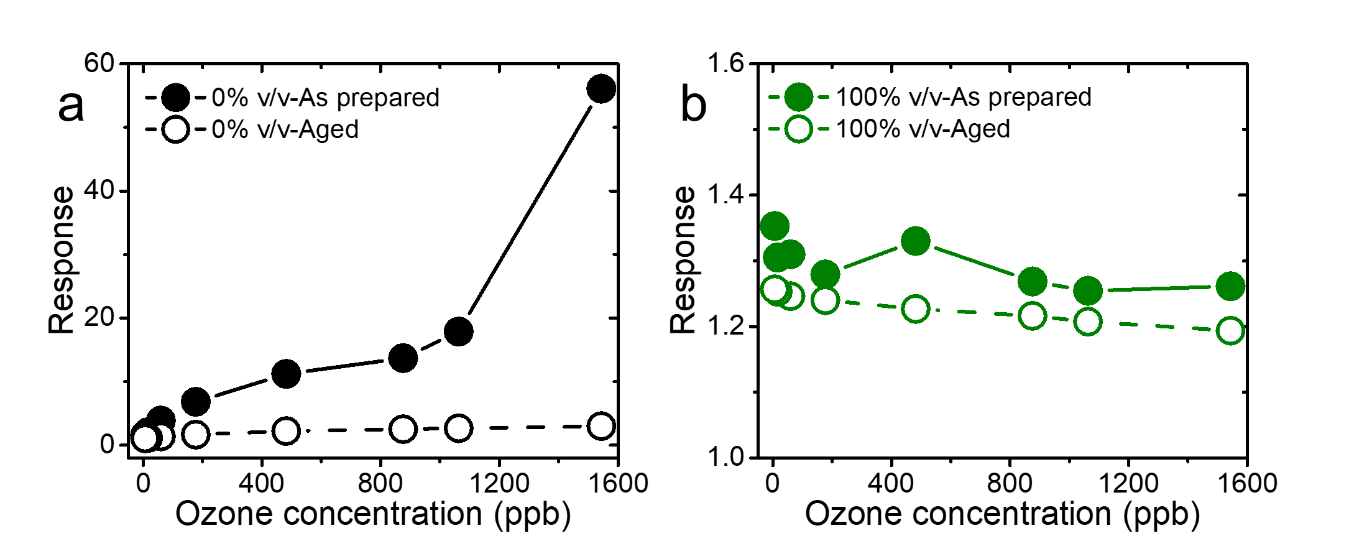
Figure S26.** Calculated response as a function of O_3_ concentration of a) 0% CsPbBr_3_ and b) 100% v/v CsPbCl_3_ perovskite-based sensors as prepared (filled circle) and after a few weeks aging (hollow circle).

**
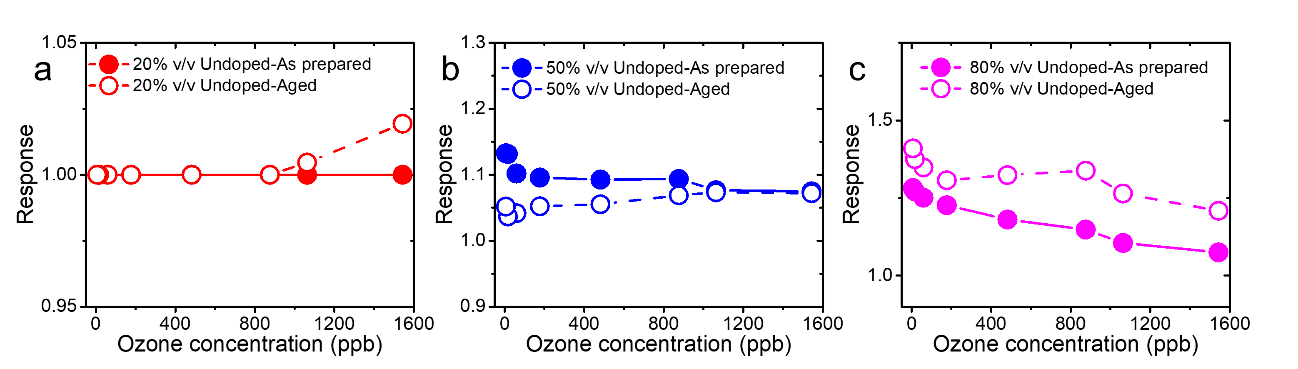
Figure S27.** Calculated response as a function of O_3_ concentration of a) 20%, b) 50% and c) 80% v/v of the as prepared (filled circle) and aged for a month (hollow circle) undoped perovskite-based sensors.

**
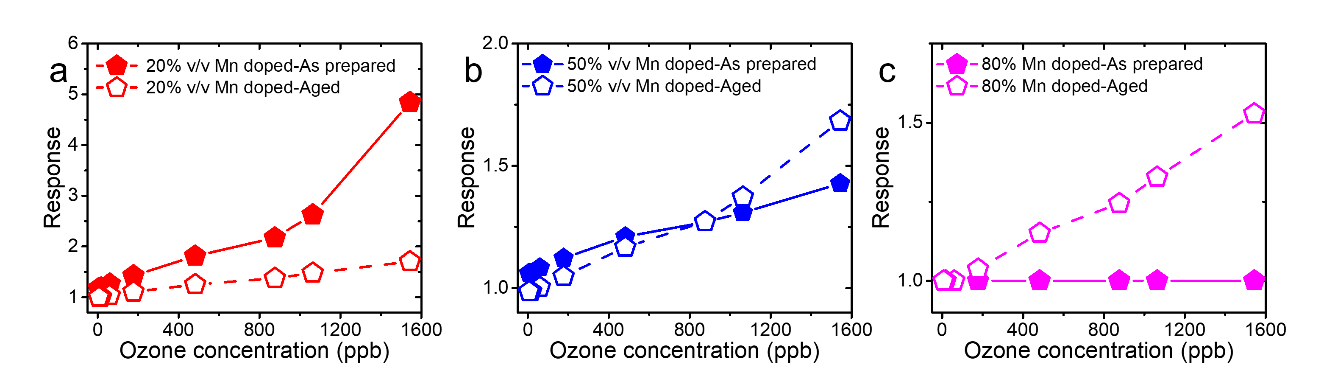
Figure S28.** Calculated response as a function of O_3_ concentration of a) 20%, b) 50% and c) 80% v/v of the as prepared (filled pentagon) and aged (hollow pentagon), Mn-doped perovskite-based sensors.

**
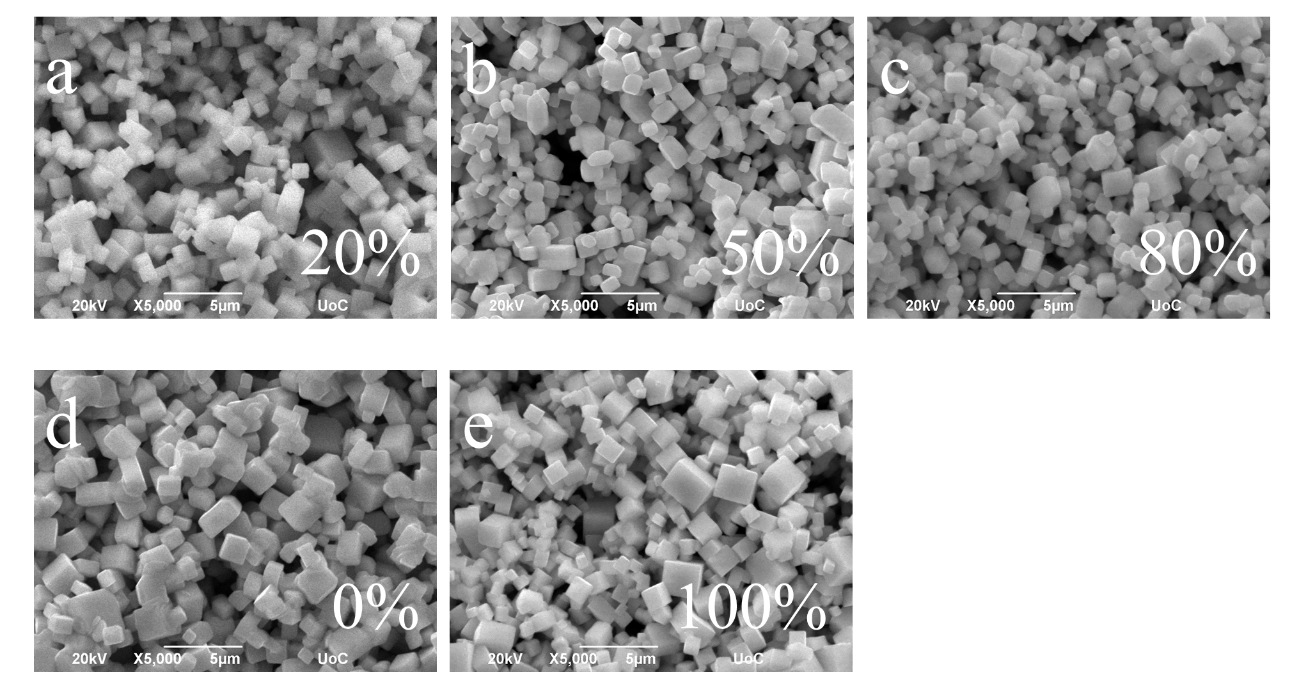
Figure S29.** SEM images of a) 20%, b) 50%, c) 80% v/v undoped mixed halide perovskite μCs and the reference samples d) 0% v/v CsPbBr_3_ and e) 100% v/v CsPbCl_3_ μCs after exposure to air for 50 days.


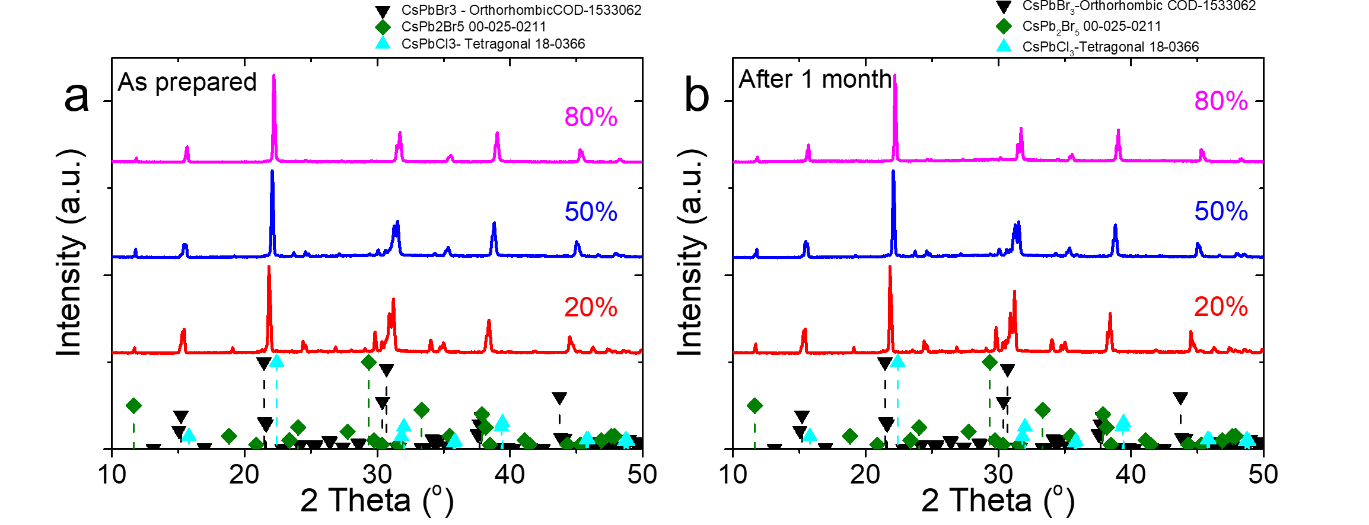


**Figure S30.** XRD patterns of 20% (red), 50% (blue) and 80% v/v (magenta) undoped mixed halide perovskites: a) as prepared and b) aged for one month.

**Figure S31.** Absorbance spectra of the as prepared (solid curves) and aged for one month (dashed curves) undoped mixed halide μCs by varying the v/v ratio.

 **Figure S32.** XPS survey spectra of the aged undoped mixed halide μCs by varying the v/v ratio.


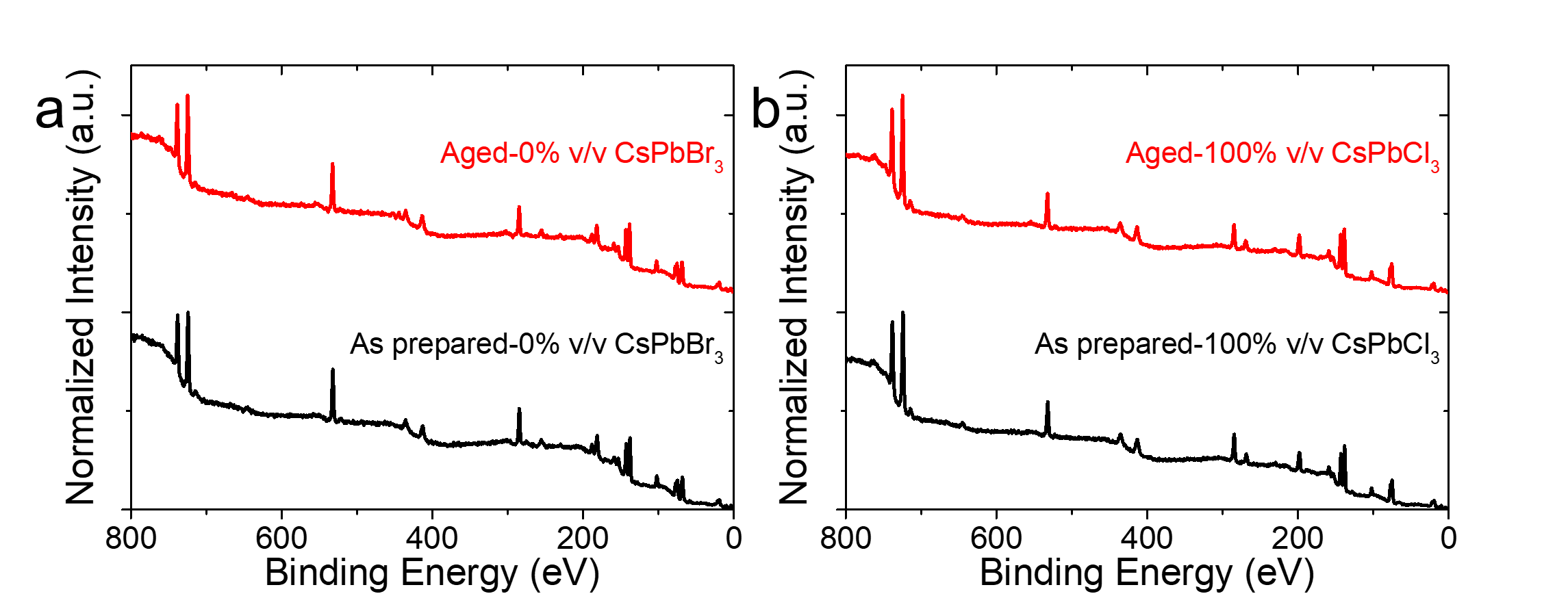
**Figure S33.** XPS survey spectra of the as prepared (black) and aged for a month exposed to ambient conditions (red) of the a) 0% v/v CsPbBr_3_ and b) 100% v/v CsPbCl_3_ μCs.


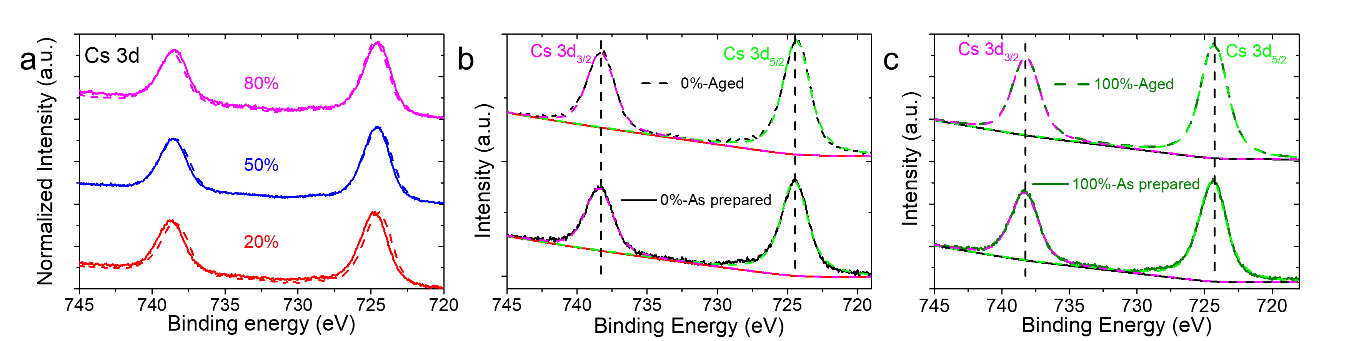
**Figure S34.** High-resolution XPS spectra of Cs 3d of the as prepared (solid curves) and aged for a month to ambient conditions (dashed curve) of the a) undoped mixed halide perovskite μCs, b) 0% v/v CsPbBr_3_ and c) 100% v/v CsPbCl_3_ μCs.


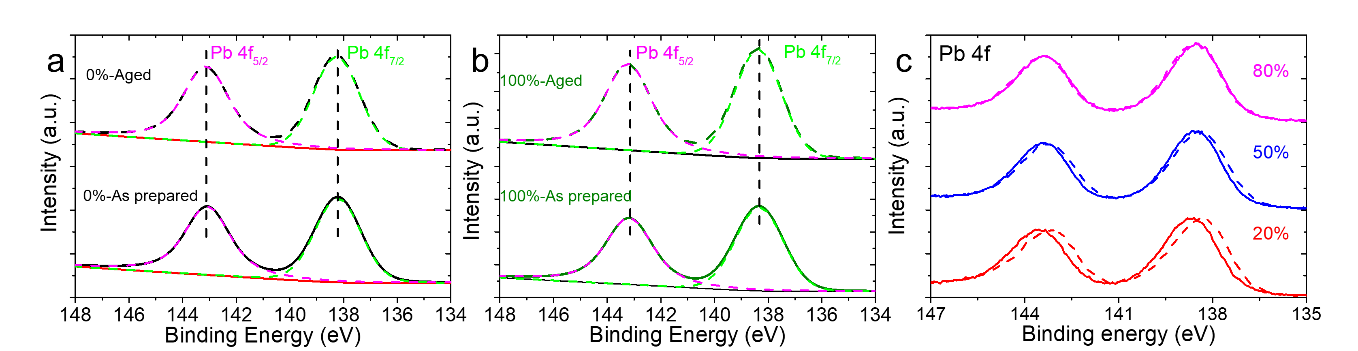
**Figure S35.** High-resolution XPS spectra of Pb 4f of the as prepared (solid curves) and aged for a month to ambient conditions (dashed curves) of the a) 0% v/v CsPbBr_3_, b) 100% v/v CsPbCl_3_ and c) undoped mixed halide perovskite, varying the v/v.


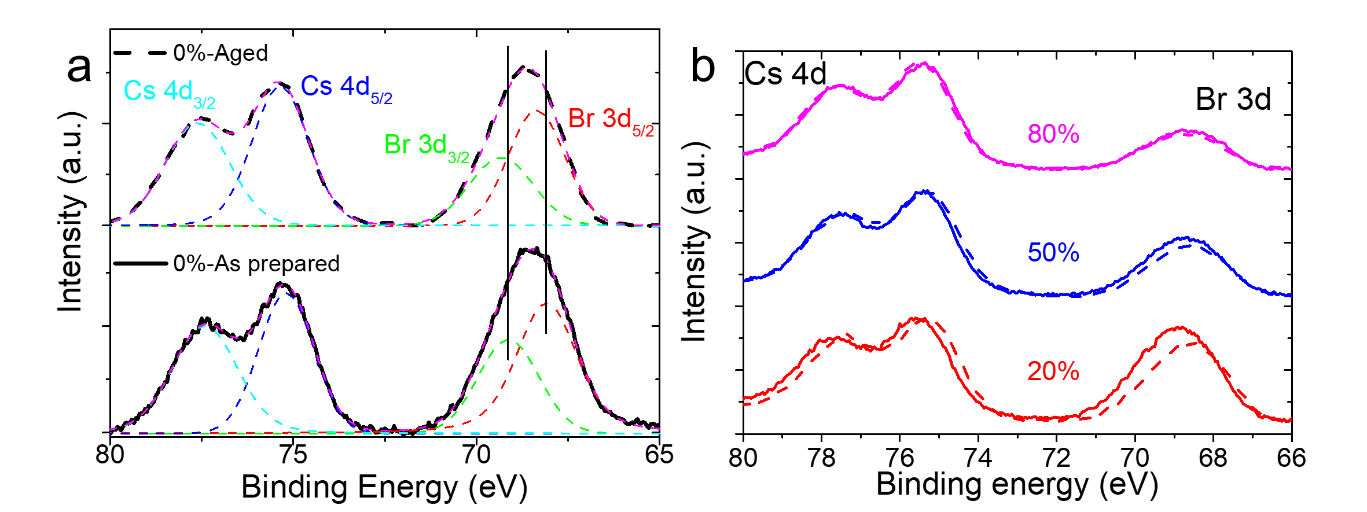
**Figure S36.** High-resolution XPS spectra of Br 3d of the as prepared (solid curves) and aged for a month to ambient conditions (dashed curves) of the a) 0% CsPbbr_3_ and b) undoped mixed halide perovskite, varying the v/v, μCs.


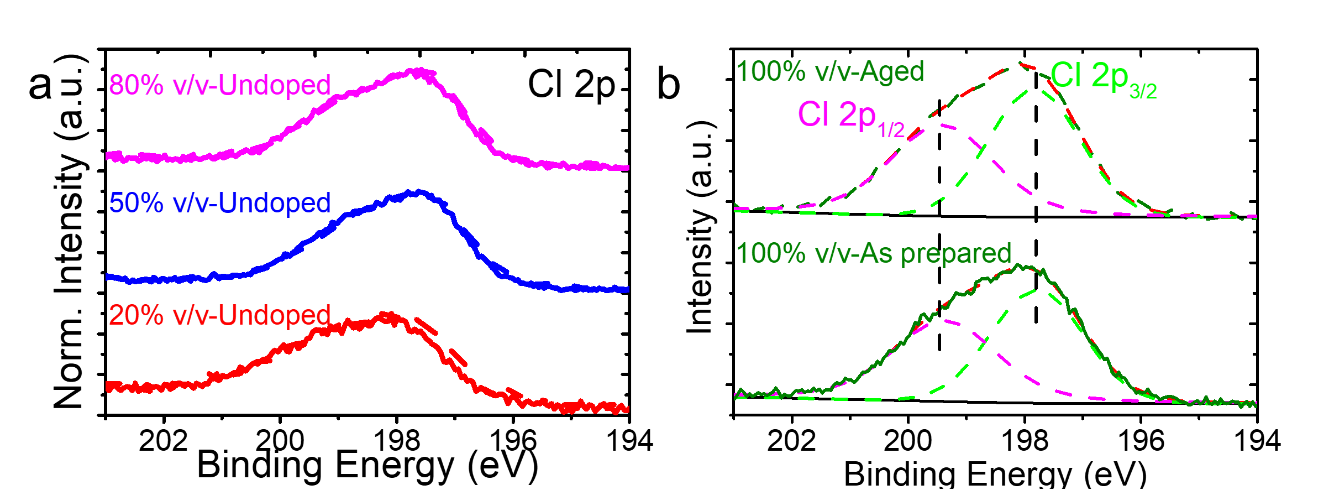


**Figure S37.** High-resolution XPS spectra of Cl 2p of the as prepared (solid curves) and aged for a month to ambient conditions (dashed curves) of the a) undoped mixed halide perovskite varying the % v/v and b) 100% CsPbCl_3_ μCs.


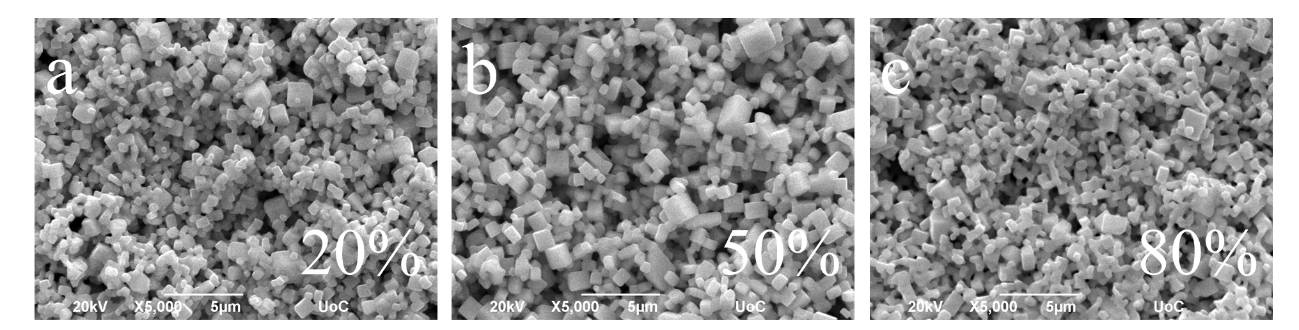
**Figure S38.** SEM images of the aged Mn-doped mixed halide perovskite μCs exposed to ambient conditions.


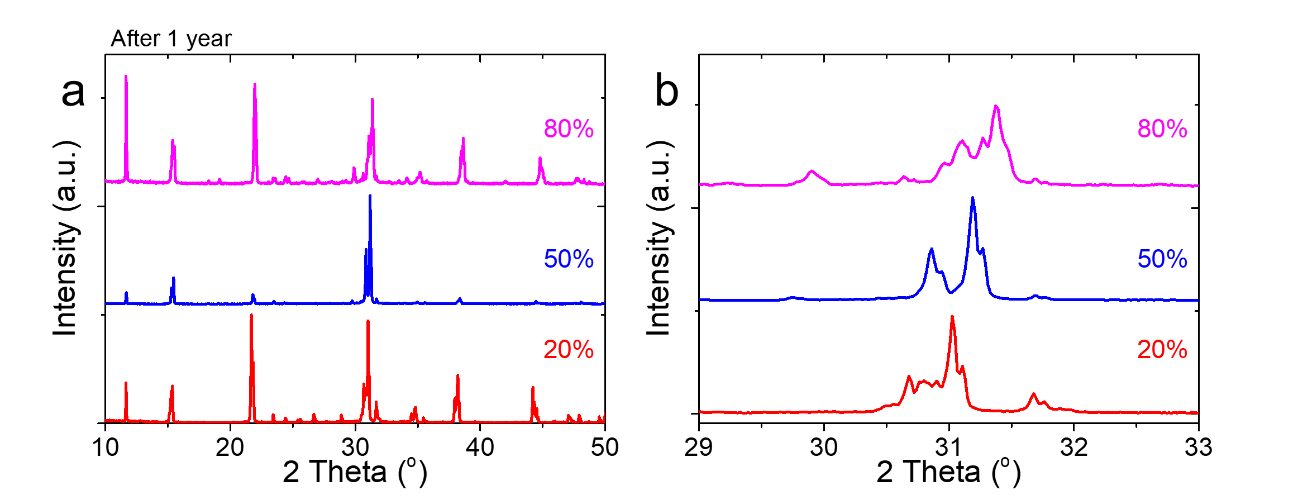
**Figure S39.** XRD patterns of Mn-doped perovskite μCs aged under ambient conditions for one year, with varying v/v ratios. XRD patterns for the 2-theta range a) from 10 to 50° and b) zooms in on the range from 29 to 33^o^.

**Figure S40.** High-resolution XPS spectra of Cs 3d of the as prepared (solid curved) and aged for a month to ambient conditions (dashed curved) Mn-doped mixed halide perovskite μCs varying the v/v ratio.

**Figure S41.** High-resolution XPS spectra of Cl 2p of the as prepared (solid curved) and aged for a month to ambient conditions (dashed curved) Mn-doped mixed halide perovskite μCs varying the v/v ratio.

**Figure S42.** High resolution XPS spectra of Br 3d of the as prepared (solid curves) and aged for a month to ambient conditions (dashed curves) Mn-doped mixed halide perovskite μCs varying the v/v ratio.

**Figure S43.** XPS survey spectra of Mn-doped mixed halide μCs by varying the v/v ratio.
